# Molecular pathways and targets in prostate cancer

**DOI:** 10.18632/oncotarget.2406

**Published:** 2014-08-29

**Authors:** Emma Shtivelman, Tomasz M. Beer, Christopher P. Evans

**Affiliations:** ^1^ Cancer Commons, Palo Alto, CA; ^2^ Oregon Health & Science University, Knight Cancer Institute, Portland, OR; ^3^ Department of Urology and Comprehensive Cancer Center, University of California Davis, Davis, CA

**Keywords:** prostate cancer, molecular targets, CRPC, localized prostate cancer

## Abstract

Prostate cancer co-opts a unique set of cellular pathways in its initiation and progression. The heterogeneity of prostate cancers is evident at earlier stages, and has led to rigorous efforts to stratify the localized prostate cancers, so that progression to advanced stages could be predicted based upon salient features of the early disease. The deregulated androgen receptor signaling is undeniably most important in the progression of the majority of prostate tumors. It is perhaps because of the primacy of the androgen receptor governed transcriptional program in prostate epithelium cells that once this program is corrupted, the consequences of the ensuing changes in activity are pleotropic and could contribute to malignancy in multiple ways. Following localized surgical and radiation therapies, 20-40% of patients will relapse and progress, and will be treated with androgen deprivation therapies. The successful development of the new agents that inhibit androgen signaling has changed the progression free survival in hormone resistant disease, but this has not changed the almost ubiquitous development of truly resistant phenotypes in advanced prostate cancer. This review summarizes the current understanding of the molecular pathways involved in localized and metastatic prostate cancer, with an emphasis on the clinical implications of the new knowledge.

## INTRODUCTION

Prostate cancer (PCa) is a complex multifaceted and biologically heterogeneous disease. The majority of men diagnosed with prostate cancer will benefit from not being treated, because they have low volume indolent tumors that do not require immediate treatment. Overtreatment of localized PCA has become a serious problem, not in the least because of serious health risks involved in prostatectomy and other commonly used approaches. Moreover, biochemical relapse occurs in about 30% of patients who were treated aggressively.

To minimize overtreatment of patients with indolent PCa, active surveillance is a reasonable and widely accepted approach [1] for many patients. The recently created and funded National Proactive Surveillance Network (NPSN), provides for this approach, and also aims to collect data and genetic sequences from biopsies to identify the molecular signatures of PCa in low-risk patients.

At the other end of the spectrum in localized PCa is the category of men presenting with either a high risk localized cancer or with metastatic disease. These are usually treated aggressively with any of the following: prostatectomy, radiation therapy and/or androgen deprivation therapies (ADT), which have been expanded in recent years to include novel substantially more efficient drugs. Nevertheless, even with newest ADT drugs, the outcome involves nearly inevitable progression to castrate resistant disease (CRPC), metastases and death. Moreover, a meta-analysis of primary ADT treatment provided conclusive evidence on the lack of survival benefit from PADT for most men with clinically localized prostate cancer [2].

The genetic landscape of prostate cancer was intensely explored in the last few years with NGS, whole genome expression analyses and analyses of epigenetic alterations. These findings, along with the results from genetically engineered mouse models (GEMM) for PCa initiation and progression revealed a number of features not encountered in their entirety in other cancers. These are:

- A relatively low rate of mutations in PCa compared to other tumors.
- Prevalence of non-random copy number variations (CNV) in most PCa tumors involving well-known prostate oncogenes or tumor suppressors.
- Recurrent chromosomal rearrangements involving ETS transcription factors, most frequently ERG, in 60 to 70% of PCa, which place these proteins under controls of an androgen-dependent promoter.
- Complex nature of the genomic rearrangements observed in PCa, with a pattern of balanced breaking and rejoining (“close chain” pattern). The highly rearranged PCa genomes are thought to evolve in a punctuated manner, with translocations and deletions occurring interdependently, via “chromoplexy” [3].
- Heavy involvement of developmental pathways that govern prostate embryonic development in the initiation and particularly progression to CRPC.
- The key role in prostate cancer of the epigenetic changes such as chromatin remodeling, DNA methylation and histone acetylation.
- The whole scale alterations in transcriptional programs, in particular those governed by androgen receptor (AR), and their prominent role in driving DNA rearrangements and co-opting developmental pathways.
- Continuum of genetic somatic changes in PCa from PIN (prostate intraepithelial neoplasia) to CRPC (castrate resistant prostate cancer), i.e. increased frequency of changes already existing in primary PCa as disease progresses to CR stage, a well as development of new somatic aberrations in CRPC.

The most common known genomic alterations in PCa involve four pathways/genes: the androgen receptor pathway, PI3K pathway, rearrangements that place members of the ETS transcription factor family under control of androgen responsive promoter TMPRSS2, and loss of function of the prostate tumor suppressor NKX3.1. The other somatic alterations and pathways involved in PCa are listed in Table 1. This review first describes somatic genetic events associated with localized disease, attempting to stratify the subtypes based on presence of ETS fusions, and describing the mutations thought to be “drivers” in the ETS fusion negative PCa. PI3K pathway involvement is also described in the section on localized PCa, even though its frequency is increased in CRPC. AR pathway aberrations in localized PCa do not typically involve AR itself, and the latter are detailed in the CRPC section.

**Table 1:** Molecular pathways in prostate cancer

Genes and alterations	Description	Alterations	Frequency in primary versus metastatic (when known)	PATHWAY
AR	Androgen receptor	Amplification Mutations Variant splicing	Only CRPC, in majority of tumors together with cofactors	Androgen receptor signaling
AR cofactors and regulators NCOA1,2,3; NCOR1, NCOR2, TNK2 and more	Regulation of the AR activity	Amplification Mutations	Infrequent in localized; 60-80% CRPC	
FOXA1	Transcription, AR co-factor, prostate development	Mutations, overexpression	5% mutations in localized, higher levels in CRPC	
Androgen synthesis enzymes: CYP17 etc	Steroidogenic/androgen synthesis	Activating mutations, copy gain	Uncommon in localized; very common in mCRPC	
TMPRSS2:ERG, other ETS	Gene fusion involving ERG; rarely other ETS family members	Translocation and overexpression	50-60% of localized and CRPC	Transcription, controlled by AR
NKX3.1	Homeobox, prostate specific, androgen regulated	Deletions	3-5% mutations, 10-20% deletions in localized, 40-80% decreased expression in CRPC	Developmental lineage specific, transcription, AR pathway
PTEN	Phosphatase suppressor of PI3K	Deletions, rare mutations	40-50% of primary, 80% CRPC	PI3K signal transduction Co-operates with AR pathway in pathogenesis of PCa
MAGI2	PTEN interactor	Rearrangement		
PIK3CA1 catalytic subunit	PIP2 kinase	Overexpression, mutations		
PHLPP1/2	Phosphatase, inhibits AKT	Deletion, down-regualtion		
Akt1	Central kinase in PI3K pathway	Point mutations (rare)		
SPOP	Speckle-type POZ domain ubiquitin ligase	Mutations	5-10% primary and metastatic	Degradation of AR cofactor NCOA3/SRC-3, and Gli factors
SPINK1	Serine peptidase inhibitor	Overexpression	5-10%, mutually exclusive with ERG rearrangements	Unknown
MYC	Master of transcription regulation; opposes NKX3.1	Overexpressed in primary, amplified in metastatic and NEPC	20-30% with gain in metastatic disease	Transcription/translation/ metabolism
NMYC	Transcriptional regulation	Overexpression, amplification	40% of neuroendocrine PCa; 5% overall	Transcription
MED12	Regulatory component of mediator complex	Mutations	2-5%	Transcription
EZH2	Polycomb group	Elevated expression	Localized (poor prognosis) and CRPC	Chromatin modification Transcriptional suppression
BMI	Polycomb group, transcriptional suppression	Elevated expression	Localized and metastatic	
TP53	Tumor suppressor	Loss, LOF*, GOF* mutations	30-100%, mostly in metastatic	Cell cycle, apoptosis, metabolism
Aurora A kinase	Mitotic kinase	Overexpression, amplification	40% of neuroendocrine PCa; 5% overall	Cell Cycle

BRAF, RAF	Serine-threonine kinases activating MAPK cascade	Rearrangements	1%, all	MAPK
CADM2	Cell adhesion molecule	Rearrangements	Primary and metastatic	Cell polarity, potential tumor suppressor
CHD1	Nucleosome positioning	Mutations	8%, mostly with SPOP mutations, in ETS normal	Chromatin remodeling
MLL complex (MLL2, ASH2L and more)	Epigenetic transcriptional activation	Mutations	9% CRPC	
TAK1/MAP3K7	TGFβ-activated kinase	Deletions	Deleted in 30% of primary and CRPC	Activation of NFkB and other not yet understood functions
RB1	Cell cycle	Loss, LOF	50% metastatic	Cell cycle
ERCC2,4,5; ATM, XRCC4, PRKDC and more	Various genes involved in DNA repair	Losses, mutations	Mostly in metastatic	DNA damage repair
CTNNB1, APC, BMP7, WNT factors	WNT developmental pathway	Losses, mutations	5% or more in CRPC	Developmental pathways
Shh, Gli factors	Hedgehog developmental pathway	Activation, elevated expression	CRPC	
SOX9	Prostate stem cells homeobox	Activation, elevated expression	CRPC	
TGFβ, TGFβR	TGFβ pathway	Activation, elevated expression	CRPC	
SMAD4	TGFβ pathway	Loss of expression	CRPC	
FGF10, FGFR	Developmental pathway, paracrine	Elevated expression	CRPC	
EGFR, IGF1R, FGFR, MET	Growth factor receptors	Activation	NA	Growth factor induced signaling, activation of PI3K and MAPK pathways, and AR signaling
IL6-IL6R	Cytokine receptor	Activation	NA	JAK-STAT3 pathway; activates AR
SRC	Tyrosine kinase	Activation	NA	Many signaling pathways
HSP90, HSP27 Clusterin/TRPM2	Maintain stability of various signaling proteins including AR and many others	Activation	NA	Protein Chaperons

* LOF; loss of function; GOF, gain of function

### Stratification of localized PCa based on molecular aberrations

Risk stratification of localized PCa is a high priority, with an overarching goal of identifying groups of patients who will benefit from aggressive treatment approaches versus those whose disease will remain indolent for years and who are good candidates for active surveillance or even no intervention at all. Ideally, it should perform better than the current histopathological/clinical grading (Gleason score in combination with tumor size, lymph node involvement, metastases, and PSA levels). However, the identification of molecular subtypes that drive differential prognoses in localized PCa was and remains a challenge.

**Figure 1:**
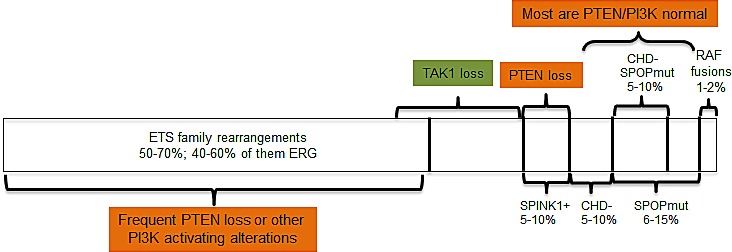
Molecular subtypes of localized prostate cancer The diagram represents the evolving understanding of the associations between molecular alterations reported in localized prostate cancer. Recent results suggest that ERG positivity and SPINK1 expression are not always mutually exclusive, and the role of TAK1 deletions in primary localized cancer remains to be explored.

Localized PC could be (relatively but not entirely arbitrary) subdivided in two categories based on presence/absence of TMPRSS2-ERG or other changes in ETS family genes (Figure 1). ETS family fusions are found in up to 60% of PCa, and the fusion-negative group could be divided into several subtypes based on results of the recent NGS studies that have identified new genetic aberrations in this group.

#### TMPRSS2-ERG and other rearrangements involving ETS family

TMPRSS2-ERG fusion [4] is a result of interchromosomal rearrangement that occurs in 40 to 60% of prostate cancers. Other members of ETS family of transcription factors, of which ERG is a member, are also involved in rearrangements, albeit much less frequently (Figure 1). This is the most frequent chromosomal rearrangement found in solid tumors, and perhaps in human cancer in general, considering the high incidence of PCa. Fusions appear to be an early event, found already in PIN, and the presence of TMPRSS2-ERG fusion is thought to be sufficient for the initiation of prostate intraepithelial neoplasia (PIN) [5]. Increased expression of ERG or other ETS factors under control of androgen responsive promoter (TMPRSS2) is an inevitable consequence of the fusion events, and it activates transcriptional program that contributes to oncogenesis by upregulating expression of, among others, MYC, EZH2 and SOX9 and repressing NKX3.1 [6-8]. The net result of high levels of ETS expression is prevention of the differentiation of prostate epithelium that is normally governed by AR.

Patients with expression of ERG in high-grade prostatic intraepithelial neoplasia are more likely to develop prostate cancer. [9]. Expression of TMPRSS2-ERG fusion shows a striking correlation with AR expression in tumor biopsies [10]. It is of significant interest that formation of fusions involving ERG genes has been shown to be facilitated by signaling from the AR, which induces proximity of the TMPRSS2 and ERG genomic loci. Both are located on chromosome 21q22, and fusion occurs via double-stranded DNA breaks [11]. In general, ERG-rearrangement positive cases contained DNA breakpoints located near AR binding sites, whereas ETS-negative prostate cancers harbored breakpoints significantly distant from AR binding sites [12]. Androgen signaling plays a direct role in generation of ERG fusions. Once the TMPRSS2 and ERG loci are rendered proximal, AR facilitates the fusion by inducing recruitment of two types of enzymatic activities - cytidine deaminase and the LINE-1 repeat-encoded ORF2 endonuclease [13]. These induce double stranded DNA breaks that are ligated by nonhomologous end joining [13]. It is also of great interest that prevalence of ETS fusions is very high in the early-onset prostate cancer (EO-PCa), defined as PCa diagnosed in patients under 50 years of age [14]. The patients with EO-PCa have higher expression of AR, and 90% of the analyzed tumors from these patients had ERG fusions and deletions of AR co-repressor NCOR2. In the older patients with lower levels of AR, structural rearrangements involved loci such as TAK1, PTEN, CHD1 that are not known to be androgen-dependent [14]. These findings indicate that AR signaling raises the probability of certain DNA rearrangements, and those involving ERG or other ETS factors and androgen responsive elements in TMPRSS2 are favored in cells with increased androgen signaling.

Because ETS transcription factors in fusion-positive tumors are expressed from an androgen-dependent promoter, their levels are significantly higher in these tumors. This is, no doubt, related to the biological role of ERG and other ETS in PCa. However, TMPRSS2-ERG expression was shown to persist in castration resistant prostate epithelial subpopulations which indicates that its expression might not be driven by androgen exclusively [15]. Presence of TMPRSS2-ERG fusion is a clear promoting event in PCa because activation of a number of oncogenic pathways is highly enriched in tumors with TMPRSS2-ERG2 rearrangement. Thus, TMPRSS2-ERG and PTEN loss cooperate in the relevant genetically engineered mouse models (GEMM) [16, 17]. TMPRSS2-ERG cooperates with activated AKT and overexpressed AR but not with loss of TP53 in transition to PCa from PIN in GEMM [18]. A conditional GEMM overexpressing ERG in prostate shows major upregulation of the AR cistrom when combined with PTEN loss [19]. Constitutively expressed ERG reprograms genome-wide localization of AR and prostate epithelium to respond to PTEN loss [19].

TMPRSS2-ERG expression induces repressive epigenetic programs by upregulating expression of the EZH2, a Polycomb group protein [20]. Overexpressed ERG in PCa shows an extraordinary degree of transcriptional co-opting of androgen receptor, with a consequence of inhibiting AR-mediated differentiation and promoting EZH2-mediated dedifferentiation [20]. Paradoxically, upregulation of EZH2 is also achieved, though by different means, in ETS fusion negative PCa (see below, under EZH2). High levels of ETS factors in fusion-positive PCa activate a transcription program characterized by enrichment of RAS-responsive elements, therefore functionally replacing activation of the RAS-MAPK pathway [21].

Deregulation of WNT and TGFβ signaling pathways was also found to be associated with TMPRSS2-ERG fusion [22]. As an oncogenic transcription factor, ERG mediates striking non-random alterations in chromatin structure thus enabling and promoting genomic rearrangements through its effects on chromatin structure [23]. On its own, presence of ETS fusions does not show striking correlations with the disease course, even though some publications have reported association with a more aggressive disease. However, overexpressed ETS proteins probably act as an “enabler” for further carcinogenic genomic changes that drive the fully transformed phenotype. In terms of prognostic significance, ERG fusions are strongly associated with high AR signaling in the early onset PCa, a particularly aggressive group of PCa, that is thought to be driven by high levels of AR [14].

Other members of ETS family were also implicated in PCa via chromosomal rearrangements. ETV1 activation in a mouse models appears to have consequences for AR transcription that are different from those induced by translocated ERG: ETV1 largely cooperates with the AR transcriptional program, and promotes autonomous testosterone production [24]. ETV1-positive tumors have a very poor outcome [24]. ETV4 is involved in translocations with TMPRSS2 in PCa less often [25], and, as seen in a GEMM, while ETV4 expression appears not to affect tumor growth per se, it induces metastatic progression in cooperation with activated PI3K pathway [26]. In human PCa, ETV4 overexpression correlates with activation of PI3K and RAS signaling [26].

##### Treatment implications

Currently there are no drugs targeting ETS family transcription factors. In preclinical studies a compound WP1130, inhibitor of deubiquinating enzyme USP9X was shown to restrain growth of prostate cancer *in vitro* and *in vivo* by promoting degradation of ERG protein [27].

It was suggested that ETS fusion positive PCa patients could benefit from treatment with poly (ADP-ribose) polymerase 1 (PARP1) inhibitors because TMPRSS2:ERG interacts in a DNA-independent manner with PARP-1 and the catalytic subunit of DNA protein kinase (DNA-PKcs). Moreover, these interactions are essential for the transcriptional program of ETS factors [28]. A randomized phase II trial NCT01576172 of PARP-1 inhibitor ABT-888/veliparib or placebo with abiraterone in fusion-positive patients with mCRPC has started to recruit patients. Another PARP-1 inhibitor, olaparib, is tested in a phase II trial NCT01682772 in UK, and this trial includes evaluation of defects in DNA repair genes in patients. A novel PARP inhibitor BMH 673 is in early testing in various tumors with DNA repair deficiencies, including PCA (NCT01286987).

#### Activation of PI3K pathway

Activation of phosphoinositide-3-kinase (PI3K) pathway, most often through PTEN copy losses occurs in 50% of PCa, and appears to be an early change, found already in PIN. PTEN is a phosphatase that is a well known as a tumor suppressor downregulating the PI3K pathway activity. PTEN deletions and/or mutations are found in 30% of primary prostate cancers [29] and 63% of metastatic prostate tissue samples [30], placing PTEN mutation among the most common genetic alterations reported in human prostate cancers. Monoallelic losses are more common in PIN and localized PCa, while bi-allelic PTEN losses are higher in frank PCa and particularly in CRPC. Moreover, homozygous loss of PTEN is causative in progression to aggressive metastatic phenotype and castration resistance [31]. ETS fusion positive tumors are enriched for PTEN loss, while the fusion-negative tumors have less frequent PTEN losses. There is a strong oncogenic interaction between high levels of ERG and PTEN loss (described above, in the TMPRSS2-ERG section).

Other components of the PI3K pathway are also infrequently altered in PCa, such as mutations in PIK3 itself, in phosphatases other than PTEN - INPP4B and PHLPP [32], or in PTEN interacting proteins MAGI2/3 [12]. MAGI proteins support the PTEN phosphatase activity and the following suppression of AKT activation. The functional relevance of these alterations remains to be verified.

Studies in GEMM strongly confirmed the role of PTEN in prostate carcinogenesis. The monoallelic ablation of PTEN in prostates of adult mice is sufficient to induce PIN that do not, however, progress to cancer [33]. These mice develop invasive tumors when genetic background includes a monoallelic inactivation of NKX3.1[34, 35]. PTEN null engineered mouse tumors are indolent and non-invasive, and additional events - such as aberrant expression of ERG [17, 36], inactivation of TP53 [37, 38] or activation of MYC [38, 39] - are needed to confer aggressive phenotype to these tumors. This could be related to the findings that loss of PTEN promotes a senescence response that prevents further development of malignant phenotype [40]. Additional alterations in PTEN deficient PCa, such as ablation of SMAD4 (key effector in TGF-β pathway) serve to overcome this senescence, leading to the development of aggressive tumors with 100% penetrance [41].

Genetic changes leading to activation of PI3K pathway through various mechanisms (PTEN copy loss, MAGI2/3 mutations, PIK3CA mutations) are enriched in tumors positive for ETS fusions. Well-supported evidence exists, mostly from GEMM, of cooperation between ETS aberrations and PIK3CA pathway in development of PCa (see above). Not much information is available about the accompanying driver mutations in a relatively small subset of T/E positive tumors with normal PI3K/PTEN status.

Aberrations of PI3K pathway contribute to development of the castration-resistance in PCa, at least in GEMM. Castration-resistant growth is an intrinsic property of Pten null prostate cancer cells, independent of cancer development stage [42]. Deletion of AR in PTEN null epithelium promoted proliferation of PTEN null cells and lead to the activation of Akt. Activated PI3K/AKT pathway is sufficient to compensate for androgen/AR-signaling blockade by inducing proliferation of basal/progenitor cells and enhancing expression of a number of pro-proliferative factors including EGR1, c-JUN, and EZH2 [42]. A recently discovered consequence of PI3K activation is accumulation of esterified cholesterol in of high-grade prostate cancer, whose significance is underlined by the finding that depletion of this form of cholesterol diminishes proliferation of PCa cells ([43].

In humans, numerous studies demonstrated the association between PTEN loss and worse prognosis, including shortened PFS [44] in particular in ERG positive cancers [45], increased risk of relapse [46] and development of metastases [47, 48].

Castration or treatment with Enzalutamide (AR antagonist) in a GEMM of high grade (HG) PIN that develops in absence of PTEN resulted in rapid progression of the otherwise stable HG-PIN to CRPC [49]. However, targeting PI3K rather than AR pathway in this model with BEZ235 (PI3K/mTOR dual inhibitor) resolved the HG-PIN phenotype. Moreover, concurrent inhibition of MAPK and PI3K in PTEN null CRPC that developed after castration was effective in inhibiting growth of these tumors. These findings have serious implications for the androgen deprivation therapies used currently for treatment of prostate cancer.

##### Treatment implications

More and more evidence suggest that ADT benefits are reduced in PCa with PI3K activation [42, 49], and that combining ADT with PI3K pathway inhibition is significantly more efficient, at least in GEMM, most likely by inhibiting the crosstalk between the two pathways. PI3K inhibitors are clinically tested in CRPC, and some trials are exploring the combination of PI3K pathway inhibition with ADT (see below, under CRPC). In a phase II trial NCT01695473 BKM120 will be given to patients with high risk PCa as a neoadjuvant, for 14 days prior to radical prostatectomy. This trial will also test the effect of this drug on activity of AKT, 4eBP and s6 kinase in the tumor samples.

A recent report on failure of mTOR inhibitor temsirolimus in mCRPC patients suggests that a single targeted therapy is not sufficient to have an impact on the course of this disease [50]. The fact that patients accrued into this trial were not pre-screened for the activation of mTOR pathway could have contributed to its failure to reach its endpoints.

#### Driver mutations in ETS fusion negative PCa

Until recently, the driver mutations in ETS fusion-negative PCa were unknown. In the last year or two, a number of genomic aberrations that occur selectively in ETS fusion-negative PCa were identified, mostly through use of NGS and analysis of epigenetic alteration.

SPOP mutations (6-15% of PCa) appear to represent a genetic subclass of PCa of its own. Mutations in SPOP are mutually exclusive with the ETS family rearrangements and rarely have accompanying mutations in PTEN or PIK3CA or TP53 in localized cancers. SPOP mutations define a subgroup of PCa with poor prognosis [51]. They are strongly associated with copy loss of CHD1/5q21.1 and copy losses of 6q21 containing loci for FOXO3 and PRDM1 [51]. In general, SPOP mutations are associated with higher frequency of CNVs. Even though SPOP mutations in localized PCa show an inverse relationship with PTEN and PI3K pathway alterations, they do co-occur more frequently in metastatic tumors [51]. SPOP is a POZ domain adaptor protein that forms a complex with CULLIN3 E3 ubiquitin ligase, and it was initially shown to ubiquitinate and induce degradation of SRC-3/AIB1, a cofactor of AR necessary for its activity [52]. This is a strong indication that SPOP loss of function deregulates activity of AR already in localized PCa. PCa-associated mutant versions of SPOP protein are unable to bind to SRC-3 and trigger its degradation [53] thereby validating the tumor suppressing role of SPOP. Recently it was shown that SPOP recognizes a degron within the hinge domain of AR and promotes degradation of AR but not of PCa associated splicing variants that lack hinge domain [54]. SPOP mutants do not activate degradation of AR [54]. SPOP also promotes degradation of Gli2 and Gli3, transcription factors in Hedgehog (Hh) developmental pathway, which contributes to castrate resistant phenotype (see below). This indicates that mutations in SPOP might lead to inappropriate activation of Hh pathway [55-57]. SPOP and Cullin3 E3 ubiquitin ligase also ubiquitinate the Polycomb group protein BMI1 [58]. Considering the role of BMI1 expression in CRPC (below), increased stability of this protein resulting from SPOP inactivation could be yet another contributor to aggressive character of SPOP mutant PCa. In addition, a single report suggested that SPOP expression might be lost in as many as 37% of PCa [59]. Therefore, SPOP is a tumor suppressor that is uniquely placed to deregulate, when mutated, the androgen signaling and three developmental pathways instrumental in prostate development and carcinogenesis.

CHD1. Loss of this chromatin remodeler occurs in 5-10 % of PCa, exclusively in ETS fusion negative tumors, and is frequently associated with mutations of SPOP [51, 60]. CHD1 might be involved in in prevention of chromosomal deletions. Loss of CHD1 in clinical specimens is significantly associated with an increased number of additional chromosomal deletions, both hemi- and homozygous, especially on 2q, 5q and 6q [61]. Inactivation of CHD1 *in vitro* prevents formation of ERG rearrangements due to impairment of androgen receptor (AR)-dependent transcription, a prerequisite for ERG translocation, which explains the mutual exclusivity of ERG rearrangements and CHD1 loss [62].

SPINK1 overexpression, found in 5-10% of PCa is mutually exclusive with ERG rearrangements [63] and strongly associated with copy loss of PTEN but normal copy number of AR in CRPC [64]. Recently, SPINK expression and ERG negative status was shown to be not mutually exclusive [65]. SPINK1 encodes a secreted serine peptidase inhibitor, Kazal type 1 that might involve EGFR in its tumorigenic effects, and defines an aggressive subtype of PCa [66]. SPINK+ETS-tumor xenografts were responsive not only to treatment with anti-SPINK1 antibody, but also to anti-EGFR antibody cetuximab, indicating a potential treatment option.

Methylation of miR-26a. ETS fusion negative PCa frequently are hypermethylated at the miR-26a locus [67]. Systematic analysis of methylated regions in fusion-positive versus fusion negative PCa revealed a much higher methylation of certain functional groups in the fusion-negative cancers, including homeobox proteins. High expression of histone methyltransferase EZH2 (see below) was implicated in this selective methylation process. The high levels of EZH2 are, in turn, a consequence of methylation of miR-26a selectively in the fusion negative PCa [68]. In early PCa, Myc negatively regulates miR-26a and miR-26b via direct binding to their promoters, and also directly activates expression of EZH2 [69].

MAP3K7/TAK1. Deletion mapping of locus 6q12-22, one of the most commonly deleted loci in PCa has narrowed it to 6q15 and identified MAP3K7 as one of five genes present within it [70]. TAK1 was deleted in 32% of 95 tumors analyzed, and deletions correlated significantly with high Gleason score. This TGF-activated kinase was proposed to be a putative prostate cancer tumor suppressor based on functional studies showing that attenuation of TAK1 expression lead to increased proliferation and metastases [71]. A very recent study involving a large number of interpretable tumors [72] showed a strong association of allelic loss of MAP3K7 with ETS rearrangement negative status of tumors, though it was found in some ERG fusion positive tumors as well. In both situations, the deletion (found in about 20% of PCa) was associated with advanced tumor stage, lymph node involvement and shortened survival. It is of interest that TAK1 was shown previously to play an essential role in the LKB1/AMPK pathway of energy sensing and, thus, in cellular metabolism [73].

### AR pathway alterations in localized PCa

The role of AR signaling in the initiation of PCa remains to be fully understood. It might depend on the nature of the initiating oncogenic signal. As an example, ablation of AR in GEMM prevents development of PIN by FGF10 signaling (paracrine), but ablation of AR in GEMM does not prevent induction of PIN by activated Akt [74].

Even though AR itself is never altered in primary PCa, about half of localized tumors harbor alterations in several of AR transcriptional cofactors/regulators [75]. Among them, NCOR2, a negative regulator of AR, is mutated in 23% of primary PCa; no increase in frequency of mutations is observed in metastatic PCa. The frequency of mutations in NCOR1 rises from 4% in primary to 16% in metastatic [75]. Activator NCOA2 is amplified in 8% of primary and 37% of metastatic, and NCOA1 in 4 and 11% respectively. Increased levels of NCO2 confer an increased AR transcriptional output even in presence of low levels of androgens. Several other cofactors and regulators of AR have been shown to be altered by copy number alterations [75].

### Other recurrent molecular aberrations in localized PCa

NKX3.1 is frequently mutated or lost in localized PCa. The current understanding of the consequences of the loss of function of this tumor suppressor will be discussed in the section on metastatic CRPC, because frequency of NKX3.1 inactivation is much higher in advanced tumors, and because it is a gene essential in developmental processes that are discussed separately below.

Classical tumor suppressors: inactivation of TP53, CDKN1B (p27/KIP), RB1 occurs infrequently in primary PCa, but is much more common in CRPC.

MED12 is mutated in 5% of prostate cancer [51]. It is a known tumor suppressor mutated in 70% of leyomyosarcomas [76], and is a component of the mediator complex. MED12 was recently found to inactivate TGFβR signaling and control response to several drugs in different cancer models [77]. Mutations of MED12 confer resistance to multiple anti-cancer therapies including conventional chemo and targeted therapies.

MYC overexpression is observed in PIN [78] and in primary PCa [79]. It has been reported that MYC is activated by the TMPRSS22-ERG rearrangement in cell culture and animal models [8]. MYC stability is regulated indirectly by the ubiquitin specific protein USP2a that is upregulated in 44% of prostate cancers [80, 81]. USP2a mediates suppression of miRNA cluster miR-34a/b and consequently upregulates MYC [82]. MYC is subject to many levels of regulation, and more the one of these are reportedly disrupted in PCa. MYC is phosphorylated and negatively regulated by PKCζ, a kinase with tumor-suppressing properties that is downregulated in some prostate tumors [83].

CADM2 is nectin-like member of the immunoglobulin-like cell adhesion molecules with expression reduced in PCa [84] and disrupted by rearrangements in 3 of 7 primary tumors sequenced and in 6 from an additional set of 90 [12]. The role of CADM2 in PCa development is not understood.

### Genetic landscape of metastatic PCA and CRPC: pathways significantly activated or deregulated compared to localized disease

CRPC is characterized by massive accumulation of genomic and epigenetic alterations involving a number of developmental, signal transduction pathways as well as oncogenes and tumor suppressor controlled pathways (Table 2). These alterations are most likely driven by the disregulated AR program and by ADT that is almost universally used in patients with aggressive and metastatic disease. Clearly, the AR program plays a critical role in PCa progression.

**Table 2:** Drug Targets in Prostate Cancer

PATHWAYS	Drug targets	DRUGS	DRUG DEVLOPMENT STAGE
AR PATHWAY	AR	Xtandi/MDV3100/enzalutamide ODM-201, ARN509	Approved Phase 3
	AR cofactors		
	Androgen synthesis enzymes: CYP17	Zytiga/abiraterone Orteronel/TAK700	Approved Phase 1/2
ETS	TMPRSS2:ERG	PARP inhibitors: ABT-888, Veliparib, BMN-673	Phase 1
Growth factor receptors	EGFR	BIBW 2992/Afatinib, Lapatinib, PLX3397	Phase II
	MET	Cabozantinib /XL184, Tivantinib ARQ 197, Onartuzumab	Phase II, III
	IGFR	Cixutumumab/IMC-A12, PLX3397	Phase I
	FGFR	Dovitinib/TKI258	Phase II
	VEGFR	Dovitinib/TKI258, Axitinib (AG013736), PLX3397	Phase I
PI3K	PIK3	BKM120, GDC0980, GSK2636771, BEZ235	Phase I
	PTEN, MAGI2, PHLPP1/2,		
	AKT1	MK2206, GDC0068	Phase I
	mTOR	Temsirolimus, Everolimus, DS-3078a	Phase I
Other kinases	SRC	Dasatinib/Sprycel/ BMS-354825	Phase I
Cell Cycle	CDKs	Dinaciclib	Phase I
	Aurora A kinase	MLN8237 (Alisertib)	Phase I
Protein Chaperons	HSP90	AT13387, STA-9090	Phase I, II
	HSP27	OGX-427	Phase II
	Clusterin/TRPM2	OGX-011/custirsen	Phase 3
Histone acetylation (transcriptional repression)	HDAC (EZH2, CHD5, MLL2)	Pracinostat SB939 Panobinostat Vorinostat	Phase I
DNA damage repair	PARP	PARP inhibitor Veliparib	Phase I
Angiogenesis	VEGFR	Dovitinib/TKI258, Axitinib (AG013736	Phase I, II
	Angiopoetin 1, 2	AMG 386/Trebananib	Phase I
Developmental pathways: NOTCH. SHH, WNT	gamma secretase	RO4929097	Phase I
	PTCH/SMOO	Vismodegib/GDC-0449, LDE-225, itraconazole	
	Wnt-5a, Fzd8	OMP-54F28, Foxy-5	

#### Androgen receptor pathway

AR pathway is a driving force in CRPC, as seen from its deregulation in vast majority of these cancers. As described above, a significant number of localized cancers have perturbations in AR associated regulators and co-factors, but not in AR [75]. However, AR itself is altered in 60% of CRPC [60, 75]. It is clear now that AR is activated in CRPC despite of castrate levels of circulating testosterone, an understanding that has driven development of the second generation of anti-androgens. In general, it is thought that the role of AR in castration resistant cancer cells is not to direct the androgen-dependent gene expression program without androgen, but rather to execute a distinct program resulting in androgen-independent growth [85].

Potential mechanisms by which AR reactivation occurs in CRPC include variable levels of AR gene amplification (30% of cases or higher), activating AR mutations, activating alternative mRNA splicing (10-25%), increased expression or activation of AR transcriptional coactivators, increased intratumoral androgen synthesis, activation of modulatory kinase pathways and noncoding RNAs (see below). All these alterations lead to sustained androgen receptor signaling in presence of castrate serum levels of androgen. The array of different mechanisms that contribute to activation of AR in CRPC is extremely diverse.

##### Aberrations in AR itself

Amplification of AR [86] occurs in about 30% of CRPC. Focal amplification of AR might predate ADT in PCA since clonal foci are found in small percentage of treatment naïve patients and are predictive of poor prognosis [87] Activating mutations are observed in 10% to 30% CRPC and confer enhanced survival in absence/low levels of androgens [88]. Treatment with antiandrogens selects for gain-of-function AR mutations with altered stability, promoter preference, or ligand specificity as shown in a number of studies [89, 90]. A striking example of the selection for AR mutations was shown in a study that sequenced AR in bone marrow metastases of CRPC developed after therapy with flutamide. Mutations were found in 5 of 16 patients, and they conferred upon AR the ability to be stimulated by flutamide [90].

More recently, a mutagenesis screen identified a mutation F876L in AR that could convert the second generation AR antagonist enzalutamide into an agonist. This works also identified compounds that could antagonize AR F876L [91]. F876L mutation was identified independently in cell lines selected for resistance to enzalutamide or ARN-509 in two other studies [92, 93], and F876L mutation was identified in plasma DNA of progressing patients [92]. These findings suggest that the potential of the long-term benefit from the second-generation antiandrogens may be reduced in the presence of resistance mutations.

Another common mode of AR activation involves alternative splicing in AR [94, 95], leading to ligand-independent activation or reduced requirement for androgens due to the lack of ligand binding domain in these variant AR proteins [96]. Alternatively spliced constitutively active AR expression is increased in cells treated with enzalutamide or abiraterone [97, 98], while the full length AR is repressed [97].

Genomic rearrangements within the AR locus were discovered that prevent expression of full-length receptor but produce truncated versions lacking the androgen binding domain. These truncated proteins maintain the AR transcriptional program constitutively and in a truly androgen independent manner [99].

- Posttranslational modifications of AR. Multiple modifications of AR by phosphorylation, sumoylation, methylation and acetylation have been reported in the literature (reviewed in [100]), many of which have consequences on AR stability and activity. Tyrosine phosphorylation of AR has been reported [101]; it appears to be accomplished by a number of different kinases [102-104] and is important for tumor growth under androgen depleted conditions.
- Somatic genetic changes in components of AR transcriptional co-regulators leading to an increased and/or changed output of AR activity. Mutational inactivation of inhibitory factors NCOR1, NCOR1 and NRIP1, and activating changes in NCOA1, NCOA2 and TNK2 are observed in primary cancers but are much more frequent in metastatic [75]. AR accessory transcription factor FOXA1 is mutated in about 5% of CRPC [60], and is described under “Developmental Pathways”.
- Intratumoral androgen synthesis is increased through elevated endogenous expression of enzymes in the androgen synthesis pathways in tumors (CYP11B1 and A1, HSD17B2, AKR1C3 and others) or conversion of circulating low affinity adrenal androgens to DHT [105-107]. Androgen deprivation promotes intratumoral synthesis of dihydrotestosterone from androgen metabolites [108]. There are suggestions that reactive inflamed prostate cancer stroma may contribute to increased intratumoral androgens [109]. Recently, the enzyme 3β-hydroxysteroid dehydrogenase type 1(3β HSD1), which catalyzes the rate-limiting step in conversion of the adrenal-derived steroid dehydroepiandrosterone to DHT, was found to be sometimes mutated in prostate cancer. The mutation N367T does not affect enzymatic activity but produces a protein resisting degradation and thus accumulating at high levels [110].
- Upregulation of AR signaling through activation of modulatory kinase pathways and AR phosphorylation. The cooperation of activated PI3K pathway in AR signaling was mentioned above in the section describing PTEN deletions. Signaling by activated Akt (as a results of PTEN loss) and ERK promote hormone-independent but AR dependent growth of PCa cells and tumors [111]. In addition, numerous publications reported that other kinases, such as Src, Pim and Aurora A are involved in progression to CRPC. Src family kinases have a tumorigenic potential in PCa in models [112, 113]. Kinase activities of EGFR, ephrin type-A receptor 2 (EPHA-2), JAK2, ABL1 and SRC are increased in PCa as seen from the analysis of the phosphotyrosine peptide enrichment [114]. The IL6-IL6R signaling leading to activation of the JAK1 - STAT3 pathway is also involved, whereby STAT3 interacts with AR and enables recruitment of p300 to AR transcriptional complex [115, 116]. Extracellular growth factors - EGF, IGF, FGF10 and others – could also lead to transactivation of AR through receptor tyrosine kinase (RTK) engagement leading to activation of PI3K and MAPK pathways. EGFR, in particular, is overexpressed in many PCa [117], and FGF receptors are involved in paracrine signaling involving modulation of AR activity (see below). MAPK pathway is frequently deregulated in metastatic PCa and CRPC and activates AR-dependent transcription [118].
- Regulation of AR degradation. Numerous publications describe multiple mechanisms of maintaining the stability of AR in CRPC. E3 ubiquitin ligases Mdm2 [119] and CHIP [120] have been implicated in the control of AR. Phosphorylation of AR by kinases could alternatively recruit ubiquitin ligases for degradation or prevent their binding for increased stability. Certain mutations in AR in CRPC serve to promote the stability of protein by modifying amino acid residues necessary for receptor ubiquitination or sumoylation and following degradation. Ubiquitin ligase Siah2 is involved in targeting for degradation a select pool of NCOR1-bound, transcriptionally-inactive AR, which promotes expression of select AR target genes implicated in lipid metabolism, cell motility, and proliferation [121].

### Treatment approaches to deregulated AR program in CRPC

#### 

##### Targeting AR, androgen synthesis and AR co-factors

AR is the primary treatment target in PCa. The development of novel therapies to achieve androgen deprivation in prostate cancer patients has improved the outlook for patients with advanced-stage and castration-resistant prostate cancer. However, in majority of patients the beneficial effects are self-limited, though some patients derive a long term or even life long benefit.

In the recent decade or so, it was realized that some CRPCs remain hormone-dependent in spite of the very low levels of circulating androgens, due to some of the mechanisms described above. That led to the development and FDA approval of the new generation of drugs such as Abiraterone (inhibitor of enzyme CYP17 in the androgen synthesis pathway) and enzalutamide (selective AR inhibitor), with more in development (Table 2). Abiraterone has a much improved efficacy compared to the “old” second line drugs, and brings significant benefits to patients with CRPC [122]. A clinical study has shown that pro-survival benefits of abiraterone are strongly associated with higher serum androgens levels at the baseline (prior to treatment) [123], however clinical benefit accrued to all patient subgroups. Therefore serum androgen measurements are not useful in prospectively selecting patients for abiraterone therapy.

TOK-001, another CYP17 inhibitor in development, not only inhibits CYP17, but also target the AR receptor itself to prevent binding of androgens or even induce AR degradation [124]. Orteronel (TAK-700) is an inhibitor of steroid 17alpha-monooxygenase in testes and adrenal gland, and has shown a promising activity in non-metastatic CRPC inducing marked and durable declines in PSA [125].

Enzalutamide has shown efficacy in CRPC patients whose disease progressed after chemotherapy [126] and in chemotherapy naïve patients whose disease progressed after ADT [127]. Similar to enzalutamide, a novel AR antagonist ARN-509 inhibits AR nuclear translocation and AR binding to androgen response elements, and has shown a promising clinical activity in CRPC [128]. ODM-201 is also an AR antagonist that facilitates formation of inactive AR complexes unable to translocate to the nucleus. ODM-201 has shown a good safety profile and activity in CRPC in a completed a phase I/II trial ([129].

Nevertheless, it is becoming apparent that ADT can activate bypass pathways that can replace AR activity in presence of AR blockade and promote anti-androgen resistance. The recent demonstration that glucocorticoid receptor (GR) is upregulated and activated in PCa models involving continuous treatment with enzalutamide and ARN-509 is a striking illustration of the adaptability of PCa to ADT [130]. Moreover, activation of GR in this setting confers resistance to enzalutamide most likely by taking over the role of AR in transcriptional output by activating a partially overlapping set of genes. This finding, if confirmed in human cancers, may lead to re-consideration of the clinical use of corticosteroids in some treatments regimens.

There is an opinion shared by a number of researchers that many prostate cancers, in particular those with deregulated signaling pathways such as PI3K, should be treated with investigational therapies that target not only AR but these signaling pathways as well [131] or with differentiation inducing therapies [132]. The PI3K inhibitors are in clinical development, and so are mTOR inhibitors, but the latter did not show much efficacy in PCa trials.

#### 

##### Therapeutic approaches to block activation of signaling pathway in CRPC

Clinical trials are ongoing that target growth factor receptors, some in combination with ADT. Phase I/II NCT00953576 explores combination of lapatinib, small molecule inhibitor of EGFR and HER2 with dutasteride, inhibitor of 5-a-reductase. Multi-RTK inhibitor sunitinib and SRC family inhibitor dasatinib are being evaluated in a randomized trial NCT01254864 with abiraterone. Dasatinib versus placebo with abiraterone is in an additional phase II trial NCT01685125. MTD of dasatinib will be given to patients undergoing ADT (abiraterone) and radiation therapy in phase I trial NCT01826838 with the hope that inhibition of SRC pathway might overcome radioresistance.

IGF1R is targeted with a humanized monoclonal antibody cixutumumab/IMC-A12. ADT (different drugs) with or without cixutumumab is tested in randomized phase II NCT01120236 for patients with newly diagnosed mCRPC. Cixutumumab is combined with mTOR inhibitor temsirolimus in phase I/II trial NCT01026623 for mCRPC.

Cabozantinib/XL184 is a multi-RTK inhibitor with activity toward MET, VEGFR2 and other RTKs. The rationale of using it in prostate and other cancers is that it could potentially inhibit the angiogenic signaling in endothelial cells and the oncogenic MET signaling in tumor cells. Recent evidence shows that cabozantinib also restrains the activity of osteoblasts therefore inhibiting growth of bone metastases in mouse models [133]. Cabozantinib indeed has shown clinical activity by improving PFS, and reducing both soft tissue and bone lesions in CRPC [134]. Currently, cabozantinib is in a dozen clinical trials for CRPC, including two phase III trials, and early phase combination trials of cabozantinib with abiraterone or other ADT drugs. Another MET inhibitor, tivantinib, is in early testing for CRPC.

Other RTK inhibitors in clinical studies for CRPC include PLX3397 (inhibitor of KIT, CSF1R and FLT3), antiangiogenic axitinib and pazopanib (VEGFR and PDGFR) and dovitinib (FGFRs and other RTKs).

#### 

##### Preclinical approaches to overcome resistance to the newer ADT drugs

Introduction of abiraterone and enzalutamide into clinical practice gave new options to CRPC patients who had none before, but development of resistance ultimately limits the impact of these agents. A recent review described some of the clinical approaches to forestall or overcome resistance to new ADT agents [135], and intense preclinical efforts are made to discover new options.

One approach relies on blocking interactions of AR with its co-activators, which are essential for the activation of the AR transcriptional program. A peptidomimetic compound was designed that selectively targets protein motif LXXLL critical for interaction of AR with co-factors such as PELP-1, and showed a promising preclinical activity [132]. A compound named EPI-001 binds to the N-terminal domain of AR that is also involved in interactions with coactivators CBP and RAP74, and inhibits AR activity causing apoptosis [136, 137]. Another compound, pyrvinium pamoate, an FDA approved anthelmintic drug, binds non-competitively to a domain of AR that is distinct from ligand binding domain, induces prostate atrophy *in vivo* [138] and maybe active in the setting of ligand independent AR signaling [139]. A recent meeting report indicated that it has activity in animal models of PCa (https://www.endocrine.org/).

Niclosamide, another anthelmintic drug approved by FDA, was identified as a potent inhibitor of variant alternatively spliced AR (AR-V7) that drives resistance to enzalutamide in prostate cancer cells [140].

The recent demonstration of preclinical efficacy of inhibiting bromodomain and extraterminal (BET) proteins in different malignancies may be applicable in CRPC. BET domain protein BRD4 was shown to interact with the N-terminal domain of AR, and the BET domain inhibitor JQ1 disrupts AR transcription program *in vitro* and inhibited growth of CRPC in mouse models *in vivo*, presenting a new epigenetic approach [141].

Targeting epigenetics turned out to be key to the activity of a compound identified initially as a active in a screen for drugs inhibiting translocations in prostate cancer. SD70 inhibits the androgen-dependent AR program, and prostate cancer cell growth, acting, at least in part, by functionally inhibiting the Jumonji domain-containing demethylase, KDM4C [142].

Based on the observation that enzalutamide resistant PCa cells exhibit increased autophagy, a study of autophagy inhibitors found that CRPC cells are sensitive to their cytotoxic action *in vitro* and *in vivo* [143].

#### PI3K/mTOR pathway and AR program in CRPC

The role of the PI3K pathway in the development of PCa and CRPC, and the reciprocal feedback regulation of PI3K and AR activities in particular gained even more importance in light of recent findings. Loss of PTEN in PCa is apparently strongly co-operative with other somatogenic changes in the development of the CRPC phenotype. A co-clinical study of GEMM with PTEN loss in prostate revealed that resistance to ADT on this background develops only in presence of additional alterations – in this scenario, loss of ZBTB7A or p53 [144]. This study conducted integrative acquisition of data from the mouse model and human PCa samples and identified changes that are associated with poor response to ADT: downregulation of XAF1, inhibitor of anti-apoptotic protein XIAP1, and upregulation of SRD5A1 (involved in the conversion of testosterone to DHT (stable form, dihydrotestosterone). Inhibition of XIAP1 with embelin administered concurrently with ADT (bicalutamide) inhibited proliferation of PCa in mice with deletion of PTEN and Zbtb7A or Pten and p53 [144]. Because corresponding changes were seen in this co-clinical study of human PCa biopsies, it is likely that combination of ADT with drugs targeting XIAP1 or SDR5A1 (dutasteride) might be of therapeutic benefit in this subset of PCa.

The second study has found that KLK4 (kallikrein regulated peptidase) and PLZF (promyelocytic leukemia zinc finger), two genes upregulated by AR, contribute to integration of AR and mTOR signaling. KLK4, long suspected as a player in PCa, apparently destabilizes PLZF through direct interaction and therefore abrogates the negative effects of PLZF on AR transcriptional activity [145]. Moreover, this abrogates the upregulation by PLZF of REDD1, a known inhibitor of mTORC1 [146], therefore suggesting that KLK4, as a molecular switch integrating AR and mTOR, is a viable target in PCa [145].

##### Therapeutic approaches to deregulated PI3K/mTOR in CRPC

Active clinical research is being undertaken to examine how inhibition of signaling pathways initiated by activated receptor kinase and mediated through the PI3K pathway might affect the course of CRPC.

Of more than 20 experimental drugs with activity against PIK3 kinase, three, BKM120, BEZ235 and GDC-0980, are currently tested in several phase II clinical trials selectively for PCa. Phase II trial NCT01385293 is recruiting patients with mCRPC for a single arm study of BKM120 at a pre-determined maximum tolerated dose. Phase Ib NCT01634061 will examine combination of either BKM120 or BEZ235 (a dual inhibitor of PI3K and mTOR) with abiraterone in patients with CRPC. Similarly, combination of BKM120 and abiraterone will be tested in NCT01634061. BEZ235 is in another multicenter trial with abiraterone, NCT01717898. Dual PI3K/mTOR inhibitor GDC-0980 is tested in a randomized phase II NCT01485861 with abiraterone. Several other PI3K inhibitors are in early clinical testing (dose escalation studies) for various cancers, including prostate.

AKT inhibitor GDC-0068 is tested in a randomized phase II trial NCT01485861 with abiraterone. The phase II randomized trial NCT01251861 testing bicalutamide alone or bicalutamide with AKT inhibitor MK2206 in patients for previously treated PCa. AKT inhibitor AZD5363 is in phase I testing, NCT01692262.

mTOR inhibitors everolimus and temsirolimus, approved for other conditions, are in early clinical testing in PCa. Combinations of temsirolimus with docetaxel (NCT01206036) and with vorinostat (NCT01174199) are in phase I testing. Everolimus with radiation treatment is explored for biochemical recurrence after prostatectomy NCT01548807, and as an add-on for patients undergoing radiation treatment with ADT NCT01642732.

#### DNA damage repair in CRPC and its association with AR activity

Defects in DNA damage repair (DDR) in CRPC. Mutations in the well-known DDR genes have been reported in CRPC and in localized aggressive cancers. Mutations of BRCA2 were identified in about 2% of sporadic PCa, but germline mutations in BRCA2 increase risk of PCa at younger age (<55 years) manifold [147]. Absolute risk of prostate cancer in BRCA2 carriers is 15% by age of 65 years, or 8.6 fold increase [148]. A different study has identified mutations and loss of BRCA2 in 12% of PCa [149]. BRCA1 has also been associated with an increased risk of sporadic PCa (3.5-fold), even though germline mutations in this gene have only been observed in 0.44% of PCa cases [150]. Germline BRCA mutations confer a particularly aggressive phenotype to PCa with a higher probability of nodal involvement and distant metastases [151]. ATM mutations and deletions were found to occur in 8% of PCa [149].

As mentioned above, the DNA damage repair (DDR) protein PARP-1 is essential for the activity of TMPRSS2-EGR in PCa [28], but also plays a major role in AR transcriptional program [152]. PARP-1 is recruited to the sites of AR targets and promotes further binding of AR; pharmacological inhibition of PARP inhibits PCa growth *in vitro* and *in vivo*.

Prostate tumors with mutated or deleted BRCA genes and ATM are candidates for treatment with PARP inhibitors in clinical trials. Several trials are ongoing, and at least two are testing PARP inhibitors in selected cancers (including PCa) with mutations in BRCA genes (phase I NCT00892736 with veliparib) and phase II NCT01078662 with olaparib.

AR and DNA damage repair crosstalk. Importantly, results from large clinical trials showed strong augmentation of efficacy of radiotherapy (RT) for aggressive PCa when combined with anti-androgen therapy, suggesting a potential role for AR inhibition in dampening DDR. Two recent independent studies elucidated the role of AR signaling in enhancing DDR. AR promotes expression and activity of key DDR factors such as DNAPK, XRCC2, and XRCC3, whereas DNAPK in turn supports the AR transcriptional program [153]. Androgen deprivation induces a decrease in transcription of key DNA damage repair genes and leads to higher levels and slow repair of DNA damage after radiation therapy, in particular non-homologous end-joining [154]. This could have an implication for the ADT effects in creating genomic instability prior to onset of castrate-resistant disease, or even contributing to development of CRPC via repression of DDR. Even in absence of DD inducing treatment, the androgen-deprived cells have a higher levels of double-strand breaks [154]. This strongly suggests that increased AR signaling promotes radioresistance.

MYB protein was found to supplant the role of AR in regulating DDR by regulating an overlapping set of genes. Knockdown of MYB or some of its targets (TOPB1, ATR, CHK1) in CRPC increased the cytotoxicity of PARP inhibitor indicating that co-targeting MYB pathway and PARP activity could be a potential treatment strategy [155].

#### Developmental pathways and genes in CRPC

Development of prostate is entirely dependent on endocrine and paracrine AR signaling, whereby expression of AR in UGS (urogenital sinus) mesenchyme orchestrates outgrowth and branching of prostatic epithelium, and the subsequent expression of AR in the epithelium is required for the production of prostatic secretion. It is now clear that developmental pathways activated by mesenchymal AR signaling and involved in the epithelial–mesenchymal interactions during prostate development could be inappropriately reactivated during tumorigenesis. These pathways are numerous (reviewed in [156], and it appears that all have been implicated in PCa, either as drivers of oncogenic transformation or, more consistently, drivers of transition to castration resistance as well as EMT (epithelial-mesenchymal transition). Indeed, deregulation of developmental pathways is usually associated with the CRPC and less so with localized tumors. The intriguing aspect of the developmental pathways deregulated in PCa is that many of them are normally active during prostate morphogenesis and branching in basal cells that are currently thought to be stem cells for all three lineages found in prostate gland.

**Figure 2:**
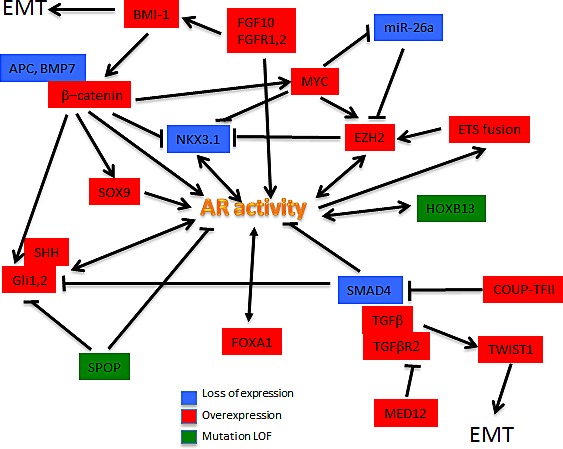
Developmental pathways deregulated in metastatic prostate cancer The schematic attempts to illustrate the complex interactions of androgen receptor with a variety of proteins with key roles in various developmental pathways.

Prostate development from UGS involves cooperation of multiple developmental pathways and gene products including but not limited to AR, SHH, FGF10, WNT, TGFβ, NKX3.1, SOX9, FOXA1 and others. Most of these appear to be involved in the development and/or progression of prostate cancer, and most interact with AR signaling (Figure 2).

NKX3.1, an androgen-regulated homeobox protein [157] is a marker of prostate stem cells; it exhibits frequent copy losses in PCa, much more frequent in CRPC versus localized disease [158]. Reduced expression of NKX3.1 might be a result of epigenetic silencing as well. NKX3.1 expression is rapidly suppressed during androgen withdrawal, a fact most likely related to the progression of the castrate resistant state.

Loss of NKX3.1 is thought to be an initiating event in prostate carcinogenesis [159]. It is mutated in one form of hereditary prostate cancer [160]. NKX3.1 and AR directly regulate each other in a regulatory loop, and, together with FOXA1 are important players in PCa progression [161]. NKXS.1 loss cooperates with PTEN loss, and Nkx3.1; Pten mutant mice develop aggressive androgen independent PCa in GEMM [35]. Interestingly, loss of PTEN causes reduced expression of NKX3.1in PCa, and functional data show that restored normal expression of NKX3.1 counteracts pro-survival and pro-proliferation effects of PTEN loos [162]. The other effects of NKX3.1 expression include increased p53 acetylation (through HDAC1) and half-life [162]. NKX3-1 copy loss is associated with an increase in genomic instability [163] and activation of MYC transcriptional program [164]. Copy loss of NKX3.1 is a strong biomarker of poor prognosis after prostatectomy or radiotherapy. When combined with MYC gain, the prognostic significance of both for biochemical relapse is even higher [163].

SOX9, similar to NKX3.1 is the early marker and an essential gene in ductal morphogenesis in prostate development [165]. In adult normal prostate its expression is found only in basal cells that are AR negative or low [166], but in PCa cells SOX9 and AR are frequently co-expressed, and SOX9 might contribute to AR regulation [166]. The oncogenic ERG expressed from the TMPRSS2:ERG fusion in PCA was shown to upregulate transcription of SOX9 in PCa by redirecting AR to a cryptic androgen-responsive enhancer in SOX9 regulatory region [6]. SOX9 cooperates in development of HG-PIN with PTEN heterozygous loss in a GEMM [167]. Deletion of Sox9 in two GEMMs of prostate tumorigenesis prevents cancer development indicating an essential role for Sox9 in PCa. [168]. Its expression is associated with higher Gleason scores and with aggressive PCa and CRPC where SOX9 activity is probably co-opted to increase growth and proliferation [167]. SOX9 might be also involved in the development of highly aggressive neuroendocrine phenotype [169].

A transcription factor ZBTB7A or LRF was implicated in regulating SOX9. ZBTB7A was unexpectedly shown to act as a tumor suppressor in prostate cancer, even though it was thought to be a proto-oncogene in other cancers [170]. ZBTB7A binds to SOX9 and antagonizes its function, and its expression is absent or low in a subset of aggressive PCa [170]. Moreover, loss of ZBTB7A cooperates with the loss of PTEN to contribute to development of CRPC phenotype in GEMM [144].

WNT pathway. β-catenin (CTNNB1) is mutated in 5% of prostate cancers [171], and mutations presumably stabilize the protein. It is essential for the identity specification in normal prostate development, but is dispensable in adult prostate maintenance [172, 173]. However, activation of β-Catenin in the adult prostate resulted in high grade PIN (HGPIN) and continuous prostatic growth after castration [174]. β-catenin is dispensable for tumor progression in the PTEN null model, but if overexpressed in this model it drives invasive growth [172]. β-catenin can directly stimulate activity of AR [175] through binding to it [176] and controls the number of progenitors in the epithelial buds and regulates a network that includes c-Myc and Nkx3.1. A small-molecule inhibitor of nuclear β-catenin activity can inhibit both the AR and β-catenin–signaling pathways in prostate cancer, and induce decreased binding of AR to its target genes sequences, as well as inhibit PCa growth *in vivo* [177]. Several other members of WNT pathway are mutated or have CNVs in CRPC. In particular, copy number losses or hypermethylation of APC, and loss of BMP7 were described [60]. The latter, a bone stroma secreted protein, suppresses bone metastases [178] and induces senescence in PCa CSCs via activation of BMP7-BMPR2-p38-NDRG1 [179]. Two agents with modulatory or inhibitory activity in WNT pathway are in early clinical testing (NCT02020291, NCT01608867; see Table 2).

Hedgehog pathway is an essential pathway in normal prostate embryogenesis [180]. Sonic hedgehog (shh) deficiency induces defects in prostate development that are due to impaired production of androgens [181]. GLI transcription factors are the main effectors of the canonical HH pathway and play an oncogenic role in a variety of cancers. The role of Hh pathway in PCa is still somewhat controversial, in part because of the widespread use of non-specific pathway inhibitors, but current results support the role of paracrine interactions versus autocrine Hh signaling in PCa [182]. Several studies have detected elevated expression of Shh and Gli2 in malignant prostate epithelium that correlated with the grade of malignancies [183, 184]. Analysis of the intermediate risk group or PCa indicated that genetic alterations in Hh pathway were associated with worse prognosis [185], and implicated serine protease inhibitor nexin 1 (PN1) as a negative regulator of Hh signaling in prostate.

The role of Hh signaling in PCa is most likely associated with its ability to modulate activity of AR [183, 186]. Hh signaling was shown to be induced in murine and human PCa following castration and to contribute to CR phenotype after ADT [187]. Hh/Gli axis supports androgen signaling in androgen deprived and androgen independent prostate cancer cells likely through a direct interaction of Gli2 with AR [188, 189]. It was suggested that Shh-Gli1 axis might govern transition form androgen-dependent to androgen independent state and even supersede the AR pathway [190]. This evidence strongly supports the role of Hh signaling in the development of castration resistance.

Hh and Notch pathways are involved in the development of resistance to docetaxel which is associated with elevated signaling and increased expression of Gli1 and Gli2 [191]. Cells with a shift to a more basal phenotype and markers of elevated Hh and Notch signaling are found in PCa biopsies and are particularly enriched in biopsies from patients who developed resistance to docetaxel based therapy. The tumors formed in a xenograft model by cells selected *in vitro* for resistance to docetaxel are sensitive to the dual inhibition of Hh and Notch pathway by cyclopamine (inhibitor of Smo) and DBZ (inhibitor of γ-secretase).

As described above, stabilization of GLI factors is one of the probable effects of SPOP mutations in PCa since SPOP participates in a pathway leading to the degradation of Gli [57]. Activated TGF-β/SMAD and WNT signal transduction pathway in CRPC also contribute to increased expression of Gli2, whereby SMAD3 in cooperation with β-catenin transcriptionally activates Gli2 [192].

##### Therapeutic implications

The SMO-targeting agents GDC-0449 and LDE225 are in Phase I/II NCT01163084 trial and entering phase I NCT02111187, respectively, for locally advanced PCA; the non-specific Hedgehog pathway inhibitor itraconazole is in phase II trial NCT01787331 for patients with biochemical relapse and in combination with orteronel in phase I/II NCT02054793 for CRPC.

TGF-β pathway and SMAD4. The role of TGF-β pathway in PCa, similar to other cancers, is complex. TGF-β is known to play a dual role in tumorigenesis, acting as a growth inhibitory tumor suppressor early in the process, and as a tumor promoter in late-stage disease. In a GEMM model of prostate tumorigenesis PTEN inactivation drives formation of indolent tumors and elicits the activation of TGF-β/BMP-SMAD4 signaling. The latter induces cellular senescence to curb tumor progression, and genetic deletion of SMAD4 (key effector in TGF-β pathway) leads to the development of highly invasive and metastatic tumors with 100% penetrance [41]. This study also verified the predictive significance of the expression signature including PTEN and SMAD4 as well as CCND1 and SPP1 (osteopontin) in a large number of PCa biopsies. Loss of expression of SMAD4 is observed earlier in PCa with high Gleason grade SMAD4, therefore, serves to inhibit PCa progression at least in early stages of tumorigenesis.

The TGF-β/SMAD4 dependent barrier to tumor progression is destructed in metastatic PCa through involvement of transcription factor COUP-TFII/NR2F2. COUP-TFII exerts its effects on TGF-β pathway by directly interacting with and inhibiting SMAD4, therefore cooperating with PTEN loss in GEMM [193]. COUP-TFII blocks the tumor-inhibiting effects of TGF-β in tumor progression to aggressive stage. Importantly, COUP-TFII is overexpressed in about 60% of prostate cancer and predicts a worsened survival [193].

In contrast to its barrier role during cancer initiation, TGFβ promotes metastatic phenotype in late stages by driving epithelial mesenchymal transition. TGFβ and TGFβR are expressed at higher levels in metastatic PCa, and are instrumental in EMT that is mediated, in part, by upregulation of the molecular chaperone clusterin via EMT transcription factor TWIST1 [194]. While SMAD3 contributes to activation of AR transcriptional activity, SMAD4, together with SMAD3 can also interact with AR and repress AR mediated transcription [195].

Radiation therapy frequently employed for treatment of PCa can increase levels of serum TGFβ and promote distant metastasis. Clinical trial NCT01427322 aims to examine if the EGFR/HER2 inhibitor lapatinib given prior to palliative irradiation for bone metastases could lower the levels of TGFβ.

Notch pathway. Notch signaling was shown to be critical for normal prostate development [196] using a conditional Notch1 gene deletion mutant. Deletion of Notch lead to enhanced epithelial proliferation in prostate, and expression of Notch1 and its effector Hey-1 gene in human prostate adenocarcinomas is significantly down-regulated compared to normal control tissue [196]. At the same time, increased Notch and Hh signaling are involved in development of resistance to Docetaxel [191]. Notch signaling also may play a pro-metastatic role by inhibiting anoikis in luminal cells [197].

Polycomb group protein EZH2. Enhancer of zeste homolog 2 is a methyltransferase and a component of repressive PRC2 complex that triggers transcriptional repression by catalyzing the addition of methyl groups onto lysine 27 of histone H3 (H3K27me2/3). EZH2 is not expressed in normal adult prostate, but is highly expressed in UGS during development and then again at puberty in prostatic epithelium [198]. EZH2 expression is high in almost all metastatic CRPC, and its expression in localized PCa is associated with poor prognosis [199]. Expression of EZH2 is negatively regulated by microRNAs miR26-a [200] and miR101 [201], of which the former is hypermethylated in ETS fusion negative PCa [67], while the latter is deleted in both localized (37%) and metastatic (67%) PCa [201]. High levels of MYC in PCa also drive expression of EZH2 by downregulating miR26-a [69]. One of the targets of EZH2 is the prostate tumor suppressor NKX3.1 [7] as well as other Homeobox genes promoters [67].

EZH2 could impede epithelial differentiation and contribute to prostate cancer progression because it was shown to directly modulate the transcriptional output of AR [202]. Moreover, EZH2, independent of its function as PRC2 component, was very recently found to act as a transcriptional activator in the deregulated AR program in PCa. Overexpression of EZH2 conferred androgen independent growth. In this setting, EZH2, together with AR, stimulated transcription from a number of genes essential for growth in androgen depleted conditions. The switch of function from repressor to co-activator was mediated through phosphorylation of EZH2 by AKT [203].

##### Therapeutic implications

Inhibitors of EZH2 GSK126 and EPZ-6438 are in clinical trials for DLCBL and FL where EZH2 is frequently mutated on Y641 and A677. Another inhibitor, 3-deazaneplanocin A, has been reported to have activity *in vitro* against PCa cells [204].

Polycomb group protein BMI1 is a member of repressive and most likely oncogenic PRC1 complex acting in epigenetic silencing of gene expression. PRC1L monoubiquitinates nucleosomal histone H2A at lysine 119 [205]. It stimulates the ubiquitin ligase activity towards H2A-K119, and is thought to exert its main role of a regulator of stem cell renewal and an oncogene in part through repression of transcription form CDKN2A locus and genes that induce senescence and cell death [206, 207]. Silencing of CDKN2A locus by BMI1 and the PRC1 complex depends on continuous presence of EZH2 [208]. Several microRNAs that are repressed by EZH2 have been shown to regulate the expression of PRC1 proteins including BMI1, indicating a coordinate regulation of PRC1 and PRC2 activities by miRNAs [209]. Bmi-1 expression is required for maintenance and self-renewal activity of prostate and PCa p63(+) stem cells and is necessary for β-catenin induced self renewal. Bmi-1 inhibition protects prostate cells from FGF10-driven hyperplasia and slows the growth of aggressive cancers with PTEN deletion [210]. Its elevated expression in PCa correlates with poor prognosis [211, 212]. Conditional overexpression of Bmi1 in mice induces PIN and promotes progression to invasive adenocarcinoma on the background of PTEN haploinsufficiency. Moreover, Akt phosphorylates and activates Bmi1 and promotes its oncogenic potential [213].

BMI1 is induced by IKKa via transcription factor E2F1 in regenerating prostate and in PCA after ADT. This is a cell-autonomous process triggered by infiltrating B cells, and links CRPC development to inflammation [214]. The regenerative response is ultimately controlled by BMI1 expression within normal or cancer progenitor cells.

HOXB13 is a homeobox transcription factor that plays a critical role in prostate development. A variant HOXB133 G84E was found to be closely associated with the risk of prostate cancer [215]. Hoxb-13 interacts with AR, and is required for full-activation of some androgen-regulated target genes [216]. Binding of HOXB13 to AR inhibits activation of genes containing androgen responsive promoter elements and activates transcription of genes containing HOXB13 response sequences [216].

FGFR pathway is intimately involved in the prostate development from urogenital sinus [217] through activation of ERK1/2, which is essential for the androgen induced morphogenesis. FGFR2 was shown to be an essential receptor in prostate morphogenesis, whose interaction with FGF10 and FGF7 control branching in developing prostate gland [218]. FGF10 functions as a mesenchymal paracrine regulator of epithelial growth in the prostate and seminal vesicle [219]. Stromal derived FGF10 stimulates growth of prostatic epithelium, and its own expression is stimulated by androgens [220]. Enhanced mesenchymal expression of FGF10 promoted formation of PIN or PCa, while inhibition of FGFR1 in epithelial compartment inhibited tumor formation [221]. Inducible expression of FGFR1 in prostate epithelium led to formation of tumors that showed characteristics of EMT and had increased expression of SOX9 and Wnt pathway receptor frizzled 4 (Fzd4), both of which are expressed at high levels in human metastatic prostate cancer [221]. In the TRAMP mouse model of PCa, mice null for FGFR1 expression in prostate cells developed smaller tumors and, more importantly, had very few metastases, while those metastases that developed had re-acquired high levels of FGFR1 and had a neuroendocrine phenotype [222].

##### Therapeutic implications

Inhibitors of FGFR signaling are in clinical trials. In particular, as FGFR was implicated in EMT and osteoblastic progression of PCa, a small molecule multikinase inhibitor dovitinib (TKI258) is explored in phase II trial for bone metastatic CRPC NCT00831792. Another inhibitor, nintedanib (BIBF1120) completed phase II trial NCT00706628, but results have not been reported.

FOXA1 is a transcription factor with a well-known essential role in prostate morphogenesis [223]. It appears to play a unique role in regulation of many nuclear steroid receptors [224], and serves as a co-factor for AR as well. Expression of FOXA1 is high in metastatic PCa [225] and is altered by copy number gain in 5% of CRPC [60]. Its role in signaling by AR is complex, as modulation of FOXA1 levels *in vitro* results in massive redistribution of AR binding sites, with some being highly enriched for AR binding, while other depleted of AR [226]. The FOXA1 deregulation leads to increased proliferation in the castrate resistant prostate cancer [227]. The precise role of FOXA1 in transcriptional transactivation by AR might have to be reconsidered in view of recent results showing that the binding sites for AR and FOXA1 identified in cell lines *in vitro* are quite different from those identified *in vivo*, with AR-FOXA1 binding diminished and AR-STAT5 binding increased [228]. FOXA1 function in promoting cell growth is AR dependent, but FOXA1 has actually an inhibitory effect on cell invasion, which is AR-independent [229]. Mutations in FOXA1 described in PCa attenuate the inhibitory effect of FOXA1 on cell motility [229].

#### Epigenetic pathways in PCa

Epigenetics encompasses several processes, such as DNA methylation, histone modifications and RNA interference. All of these are altered in PCa initiation and more so during progression, and play a functional role in prostate carcinogenesis. The role of epigenetic deregulation is PCa and particularly CRPC is strongly supported by a number of somatogenic alterations in multiple genes whose products are involved in DNA or histone modifications. These alterations result in whole-scale changes in DNA methylation and histone acetylation. Historically, hypermethylation of DNA has been long known to occur in PCa, with GSTP1 discovered as one of the prominent targets [230]. Regions that are frequently hypermethylated across individual tumors tend to be markedly enriched for cancer- and development/differentiation-related genes including tumor suppressors [231]. Aberrant methylation has been shown to be associated already with benign prostate hyperplasia and specific changes are found during PCa progression (reviewed in [232]. Methylation of CG islands was shown to increase with disease progression from benign hyperplasia to CRPC [233]. Importantly, detection of methylation could be used in diagnostic and prognostic procedures. Recent evidence suggests that hypermethylation of some genes including GSTP1 may be not causative in gene repression but rather be a consequence of differentiation and hyperproliferation of cancer cells ([234].

Intercommunication of paracrine signaling and epigenetic alterations was demonstrated in a number of studies, where targeted inactivation of TGFβ receptor [235] or overexpression of FGF [221] in mesenchymal compartment lead to development of PIN. Overexpression of chromatin remodeling protein Hmg2a in stromal cells was sufficient to induce dramatic hyperplasia and multifocal prostatic intraepithelial neoplasia in the adjacent naïve epithelial cells [236]. This striking effect was mediated by paracrine Wnt-dependent signaling, and was further promoted towards frank prostate cancer by enhanced expression of AR in stroma.

Activation of AR transcriptional program in CRPC apparently involves numerous chromatin remodeling events. For example, genomic studies have recently revealed that AR might act as a global transcriptional repressor. In embryonic stem cells, androgen-responsive elements (ARE) in AR-repressed genes are occupied by repressive Polycomb group protein EZH2 that maintains the undifferentiated state. These genes are also silenced in castration-resistant prostate cancer conferring to them a stem cell de-differentiated phenotype and promoting tumor progression [237]. Transcriptional program of AR in PCa cells involves acetyltransferase p300 that is required at an early stage of chromatin remodeling and transcription complex assembly after binding of androgen receptor [238].

EZH2 and BMI1, already described above, are prime examples of how components of chromatin remodeling complexes are involved both in development of prostate and in PCa. CHD1, frequently inactivated in PCa [33, 61], is also global chromatin remodeling factor. In addition to CHD1, other components of the MLL (mixed-lineage leukemia) complex are affected by mutations or CNV in CRPC. In particular, MLL2 and ASH2L directly interact with AR, and mutations are found in MLL2 (9% of CRPC), ASH2L, UTX, and ASXL1 [60].

The chromatin-remodeling complex SWI/SNF plays a tumor suppressor role in PCa which is antagonized by a long noncoding RNA SChLAP1 [239]. In particular, SChLAP1 reduces chromatin binding of SNF5, a key subunit of the complex, and deregulates transcription of SNF5 target genes. SChLAP1 is overexpressed in about 25% of PCa and is a strong predictor of recurrence and mortality [239].

#### Cell cycle regulation/tumor suppressors/oncogenes and other

Inactivation of tumor suppressors TP53 and RB1 is much more common in CRPC than in localized cancers [240-242]. Loss of RB1 function through various mechanisms was observed in PCa, and is associated with late stages and particularly CRPC [243, 244]. RB1 might control androgen signaling and progression to castrate-resistant phenotype [245, 246]. CDKN1B (p27KIP) is also deleted or mutated in PCa [51].

A recent study suggests that metastasis suppressor p63 inhibits EMT and metastases at least in part via regulation of miR-205. Either or both p63 and miR-205 are absent in lymph nodes or distant metastases of PCa patients [247].

MYC amplification is very common in CRPC, found in at least a third of these tumors. As mentioned earlier, MYC is overexpressed already in PIN but amplifications are mostly limited to CRPC [78]. C-Myc and Pim1 activation in GEMM induces neuroendocrine type of PC [248]. KRAS, ROS1, and MET mutations, though rare, are found at higher rate in mCRPC than in primary cancers; CDK4 is more frequently amplified in mCRPC.

RAF. Rearrangements of RAF oncogenes (SLC45A-3-BRAF, ESRP1-RAF1) are found in 1-2% of PCa, mostly CRPC [249]. These rare tumors are of clinical interest because they could be potentially targeted with inhibitors of RAF, BRAF and MEK.

UBE2C is overexpressed in many tumors, including CRPC [250]. UBE2C is an anaphase-promoting complex/cyclosome (APC/C)-specific E2 ubiquitin-conjugating enzyme, and upregulation of UBE2C inactivates the M phase cell cycle checkpoint [251]. Activation of UBE2C expression involves binding of the PI3K/AKT phosphorylated co-activator MED1 to the long range UBE2C enhancers, and chromatin looping through recruitment of FoxA1 [252]. Expression of UBE2C is driven by androgen receptor [85]. Epigenetic marks at the UBE2C enhancer, notably histone H3K4 methylation and FoxA1 binding are present in androgen-independent cells, and they direct AR-enhancer binding and UBE2C activation [85]. Increased expression of two constitutively active AR splice variants driven by treatment with abiraterone or enzalutamide was accompanied by increased expression of UBE2C, and expression of these variant but not full length AR positively correlated with UBE2C in clinical CRPC specimens [97]. Therefore, expression of UBE2C could contribute to drug resistance to CRPC therapy.

Estrogen receptor β. Estrogens were originally used to treat PCa to reduce the hypothalamic pituitary stimulation of LH/FSH production and further reduce the synthesis of androgens in 1942, reprinted in [253]. However, stimulation of ERβ has a number of serious clinical consequences and the use of estrogens was eventually replaced by other methods of achieving chemical castration, even though recently there has been a renewed interest in their use for PCa. Expression of ERβ in prostate was described in 1996 [254], and in PCa it correlates inversely with the Gleason grade [255]. ERβ, as opposed to ERα, is thought to have anti-proliferative, pro-apoptotic and anti-metastatic properties in cancer in general and PCa in particular, and could be an actionable therapeutic target in PCa (reviewed in [256, 257]). Several selective ERβ agonists have been discovered or synthesized (reviewed in [258]), including some of botanical origin, or phytoestrogens [259].

ERβ agonist induces apoptosis in prostatic stromal, luminal and castrate-resistant basal epithelial cells in BPH of estrogen-deficient aromatase knock-out mice, as well as in xenografts of prostate cancer. ERβ is downregulated in high grade PCa via TGFβ and hypoxia, and loss of ERβ is sufficient to promote EMT in PCa. ERβ expression induces destabilization of HIF-1α and transcriptional repression of VEGF-A [260]. The mechanism of the destabilization of HIF-1α involves direct transcriptional activation of prolyl hydroxylase 2 (PHD2) by ER. PHD2 is a 2-oxoglutarate-dependent dioxygenase that hydroxylates HIF-1α and targets it for recognition by the von Hippel-Lindau tumor suppressor and consequent degradation. PHD2 is activated by ERβ in a ligand-dependent manner and contributes to maintenance of the epithelial differentiation [261].

ERβ agonist treatment attenuates clonogenicity and self-renewal of murine prostatic progenitor cells and depletes both murine and human prostatic basal cells. Subsequent to castration ERβ induces further apoptosis in basal, luminal and intermediate cells [262].

ERβ ligands are not currently in clinical development for PCa, either as monotherapy or in combination with ADT.

### Prostate cancer stem cells

Much controversy existed and some still exists in the field concerning the nature of prostate and PCa stem cells. The prostate gland contains epithelial luminal cells with high levels of expression of AR, basal cells with low or absent AR and rare neuroendocrine cells. There is an agreement regarding the origin of prostate stem cells, which have been amply demonstrated to reside in the basal multipotent population [263-266]. A subpopulation of basal cells expressing high levels of TROP2 was shown to be able to form spheres *in vitro* and give rise *in vivo* to basal, luminal and neuroendocrine cells thus exhibiting multipotency [267]. However, androgen-induced regeneration after castration is mediated by both basal and luminal progenitors rather than by multipotent stem cells [268, 269]. Thus, basal cells were traced to give rise only to basal cells during prostate regeneration *in vivo*, and luminal cells produce only luminal cells under these circumstances, with both lineages self-sustained in the normal adult prostate [268].

Lineage-tracing approaches have identified rare luminal cells, named castration-resistant Nkx3-1-expressing cells, or CARNs, as also possessing multipotent stem cell activity [269]. However, the stemness of rare luminal cells in normal prostate was not confirmed in other studies [268, 270].

In terms of PCa initiation, evidence exists that supports involvement of both lineages. Prostate luminal cells, transit-amplifying cells (that have characteristics of both basal and luminal cells), and basal cells have all been implicated as the cells of origin for prostate cancer. Basal cells with introduced relevant mutations demonstrated ability to form PCa thus supporting their initiating role in PCa. The tumor-initiating ability was found to reside in basal cells CD49fhiTrop2hi expressing p63 [263, 271].

At the same time, the rare luminal cells, CARN, could also give rise to PCa on a PTEN null background [269]. In lineage tracing during PCa initiation on PTEN null background, it was found that in basal cells PTEN induces differentiation into luminal cells, and this has been shown to be an essential step for disease initiation in this model [268]. Disrupting the tumor suppressor Pten in luminal cells also led to prostate cancer initiation, with a faster dynamic compared to basal cells populations. A recent publication described a bipotential basal progenitor that can give rise to luminal cells with transit-amplifying characteristics [272]. The oncogenic transformation of basal cells promoted luminal differentiation of their progeny. This study also proposed that prostate tumors arising from luminal cells based on gene expression signature are more aggressive and have a worse prognosis [272]. Yet another study delineated different contributions of basal versus luminal cells to initiation versus maintenance and progression of PCA. It concluded that while basal cells are the initiating cells in PCA, “advanced prostate adenocarcinoma initiated in basal cells can be maintained by luminal-like tumor-propagating cells” [273].

The cancer stem cells (CSC) of basal or liminal origin are very likely a source of treatment-resistant cells. The studies mentioned above described castration-resistant cells in both CSC populations. The prostate CSC do not express androgen receptor, or have very low levels of it, and therefore survive the androgen deprivation serving as a reservoir of treatment-resistant cells [274]. These putative androgen receptor negative cancer stem cells are likely to be resistant to most androgen-based therapies, contributing to the evolution of castration-resistant disease.

To support this notion, a cell population characterized by low levels of PSA (PSA(-/lo)) was identified as being quiescent, refractory to androgen deprivation, having high clonogenic potential and long-term tumor-propagating capacity. These express stem cell genes and can undergo asymmetric cell division to generate PSA(+) cells. PSA(-/lo) PCa cells resist androgen ablation in castrated hosts, and they harbor highly tumorigenic castration-resistant PCa cells. PSA(-/lo) cells may represent a critical source of castration-resistant PCa cells [275].

Apparently, some phenotypic markers of PCa stem cells might have a functional significance in development of PCa. Trop2hi has been shown to play a significant role in stem cell renewal and epithelial hyperplasia via β-catenin pathway. Trop2 undergoes intramembrane proteolysis to release two polypeptides, of which the intracellular one translocates to the nucleus. High expression of the Trop2 intracellular domain promotes self-renewal through signaling via β-catenin and is sufficient to initiate precursor lesions to prostate cancer *in vivo* [273].

The role of the Polycomb group protein BMI in regeneration of normal prostate progenitor cells and in PCSC was described above. A recent publication elucidated a signaling axis involved in both normal prostate regeneration and in emergence of CRPC after ADT that consists of IKKa-E2F1-BMI1. Nuclear IKKa controls CRPC development through expansion of BMI1+ progenitors. The most intriguing aspect of these findings is that expression of BMI1 is triggered by inflammation that depends on the infiltration of B cells into regenerating prostate rudiments, either normal or cancerous, after ADT. The BMI1 controlled tumor growth is therefore at least partially cell-autonomous [214].

Integrin β4 was shown recently to promote self renewal of putative cancer stem cells that are basal in origin. β4 promotes adhesion of the cells to the basal membrane, which apparently is necessary for the maintenance of stemness. More importantly, mutation of β4 prevents tumor formation on PTEN null background. Finally, the high level of expression of integrin β4 in prostate cancers was associated with androgen independent metastases to bone. Finally, integrin β4 is associated with activation of Erbb2 and Met receptor tyrosine kinases, and pharmacological inhibition of these results in efficient inhibition of tumor growth in mice [276]. This indicates that combination of lapatinib and cabozantinib could have promise in treatment of PCa.

Recently, a report was published that described establishment of a xenograft model capable of supporting growth of stroma-supported xenografts from multiple patients with early stage disease [277]. More importantly, the model allows to follow the fate of tumor cells that survive after castration, therefore it might be used for the identification of castrate resistant PCa cells that are responsible for the emergence of CRPC [277].

### Epithelial-Mesenchymal transition (EMT)

EMT endows cells with migratory and invasive properties, induces stem cell properties, and prevents apoptosis and senescence, thus orchestrating the initiation of metastasis. EMT is characterized by the loss of expression of E-cadherin and induction of N-cadherin, loss of cell polarity and dependence on adhesion, all contributing to metastatic phenotype. Numerous pathways have been implicated in EMT in PCa, including some developmental pathways, inflammation driven signaling, ERG fusions and others, some of which are listed below.

Androgen deprivation induces expression of N-cadherin and EMT [278] *in vitro* and in patients. This transition was observed in normal prostate upon ADT and in PCa patients treated with ADT, and involves transcription factor ZEB1 [279]. In addition, upregulation of ZEB proteins is induced by several growth factors such as IGF-1 [280] and PDGF-β [281] that promote EMT *in vitro*.

EZH2 can induce EMT and increase the metastatic potential of prostate cancer cells by downregulation of DAB2IP, a tumor-suppressive Ras GTPase-activating protein (RasGAP) [282, 283]. EZH2 is, in turn, regulated by SOX4 [284], a homeobox transcription factor that was shown to act as an oncogene in PCa based on its overexpression and essential role in survival of PCA *in vitro* [285]. SOX4 appears to be a master regulator of EMT primarily through upregulating EZH2 expression in breast cancer [284].

TMPRSS2/ERG was also shown to promote EMT via direct transcriptional activation of expression of ZEB1, and indirect activation of ZEB2 through IL1R2 and SPINT1 [286]. In addition, ERG induces loss of cell adhesion by activating the WNT pathways through FZD4 to induce EMT and loss of cell adhesion [287].

TGF-β represents a potent EMT inducer in normal development and tumor progression via Smad-dependent and independent transcriptional pathways [288]. Smad-mediated induction of Snail, Slug, and Twist via high motility group A2 (HMGA2) and Smad-independent phosphorylation of Par6 contribute to dissolution of cell junction complexes. TGF-β also induces expression of clusterin, a pleotropic chaperone protein [289] through activation of TWIST1 [194], a known inducer of EMT. Interestingly, another chaperone protein HSP90, in its secreted form, was shown to be involved in EMT of PCa cells *in vitro* and in patients [290]. TWIST1 is upregulated by enzalutamide treatment along with activation of PKC, and both could be reversed by addition of PKC inhibitor Ro31-8220, at least *in vitro* [291], suggesting a potential approach to overcoming EMT associated with androgen deprivation.

Monoamine oxidase A (MAOA), a mitochondria-bound enzyme, was recently implicated in EMT in PCa. MAOA catalyzes the degradation of monoamine neurotransmitters and dietary amines producing peroxide as a by-product and increasing levels of cellular ROS. Expression of MAOA is associated with high grade PCa [292], and causes activation of VEGF and its co-receptor neuropilin-1 which in turn, promotes AKT/FOXO1/TWIST1 signaling and EMT. Monoamine oxidase inhibitors were the first antidepressant drugs in use, and one of them, chlorgylin, a selective MAOA inhibitor, blocked PCa growth *in vitro* and metastasis *in vivo* by disrupting the signaling leading to oxidative stress, hypoxia and EMT [293].

Expression of inducible FGFR1 in a mouse model induces PCa with EMT characteristics and involves activation of SOX9 transcriptional activity and activation of WNT pathway protein Fzd4; this was validated in human PCa [294].

β2-microglobulin is a pleiotropic signaling molecule that is highly expressed in bone metastases in PCa. β2-M interacts, among many other proteins, with hemochromatosis protein HFE, modulating iron homeostasis and leading to activation of HIF-1 (hypoxia-inducible factor-1) signaling pathways [295]. HIF1 activates the expression of number of genes including VEGF that have been linked to EMT transition *in vitro* [296]. Estrogen receptor β inhibits EMT in PCa cells by destabilizing HIF-1α and inhibiting VEGF-mediated nuclear localization of SNAIL [260].

Paracrine interactions are also contributing to EMT in PCa. One mechanism involves tumor secreted IL-6 that elicits secretion of metalloproteases by stroma [297]. Cancer associated fibroblasts develop an inflammatory signature characterized by activation of COX-2/NF-κB /HIF-1, which induces generation of reactive oxygen species and the EMT program in prostate cancer cells [298].

Another example of the role that paracrine interactions play in EMT and metastasis of PCa involves tumor secreted cytokine CXCL16 and its receptor CXCR6 expressed on bone marrow derived mesenchymal stem cells (MSC). CXCR6 induced signaling on MSC promotes their recruitment into PCa tumors, conversion into cancer – associate fibroblasts (CAF) and secretion of CXCL12/stroma derived factor 1. In turn, CXCL12 stimulates PCa cells expressing the CXCL12 receptor, CXCR4, which facilitates EMT, migration and metastasis [299].

EMT might play a critical role in the metastatic behavior of PCa, in particular bone metastases that present a very serious clinical challenge. Some investigational agents that might show efficacy in the spread of bone metastases are listed in Table 3, and they include cabozantinib, an inhibitor of MET, an RTK with a well know role in EMT. Cabozantinib shows anti-metastatic activity in mouse models of bone metastases [133] and significant clinical activity in patients with bone metastatic cancer [134]. Inhibitors of FGFR signaling (dovitinib) are also in clinical trials, as described above. In 2013 FDA approved a monoclonal antibody agent for the treatment of bone metastasis Denosumab (Xgeva), inhibitor of receptor activator of nuclear factor kappa beta ligand (RANKL) that was shown to delayed skeletal events. Denosumab was also shown recently to modestly prolong time to bone metastases in patients with non-metastatic disease [300], however the FDA did not approve it for this indication as there was no an associated survival advantage. Also in 2013 FDA approved radium-223 chloride (Xofigo) for the treatment of mCRPC patients whose metastases are primarily limited to the bones. Radium-223 is an alpha-emitting alkaline earth metal ion, which, similar to calcium-ions, accumulates in the bone. Radium-223 therapy modestly extends OS and delays the occurrence of skeletal complications of prostate cancer.

**Table 3:** Targeting bone metastases

DRUGS	Description	Stage of development	Other Notes
Cabozantinib/XL184	Inhibitor of MET and other RTKs	Promising results from Phase 2 In phase II trial with abiraterone and enzalutamide	Observed reduction of soft tissue lesions, improvement in PFS, resolution of bone scans
Denosumab /XGEVA	Receptor activator of nuclear factor-kappa B (RANK) ligand, RANKL	Approved by FDA in 2013	Superior to the previously tested zoledronic acid
PLX3397	Multitargeted inhibitor of receptor tyrosine kinase of KIT, CSF1R and FLT3 (mixture of inhibitors)	Phase 2	
Alpharadin^®^ (Radium-223 dichloride)	Short-lived alpha-particle-emitting radium-223 localizes to bone metastases and kills tumor cells	Approved by FDA 2013	In phase II-III trials in combination with ADT
Enzalutamide (MDV3100) in Combination With Abiraterone Acetate	To achieve a more complete inhibition of AR signaling via inhibition of both CYP17 and AR	Phase 2	

### Neuroendocrine PCa (NEPC)

NEPC is a subtype in a poorly defined group of prostate cancers that are variously described as “anaplastic”, “small cells PCa”, or simply “aggressive” and may represent different histopathological entities. These are associated with at least one of the following characteristics: exclusive visceral metastases, or predominantly lytic bone metastases, bulky tumors, low prostate-specific antigen, lack of or short response to androgen deprivation therapy and good but short-lived responses to platinum-based chemotherapy [301]. Classic NEPC subtype do not express AR and thus do not respond to ADT These “aggressive” prostate cancers rarely arise “de novo”, and most often appear after ADT, at a frequency of 10 to 20% [301]. An even higher proportion of CRPC demonstrate a mixed histology with features of neuroendocrine differentiation [302].

It has been suggested that introduction of the new ADT agents abiraterone and enzalutamide has significantly increased the emergence of castrate-resistant cancers with neuroendocrine features and visceral metastases [303-305]. The frequency of NEPC, a resistant form of PCa, is indeed on the rise, but the reasons for this increase are under intense discussion and are not yet resolved [306]. It is accepted that development of the aggressive NEPC phenotype is generally treatment-related, i.e. it is strongly associated with the development of castrate resistance [307]. NEPC tumors do not express AR or PSA, and comprises only about 0.5 to 2% of untreated PCa. NEPC express neuroendocrine markers, respond poorly to treatment and metastasize to visceral organs such as liver.

A first-in-class xenograft model established with xenografts from the fine-needle biopsies showed that neuroendocrine PCa can evolve directly from adenocarcinoma via an adaptive response after prolonged exposure to androgen withdrawal [308]. Possible molecular changes contributing to NEPC were analyzed in mouse models and by next generation RNA sequencing. Data from GEMM implicated the ubiquitin ligase Siah2 which regulates HIF1α degradation in the development of neuroendocrine phenotype [169]. In particular, HIF1α and FoxA2-regulated genes Hes6, Sox9 and Jmjd1a are involved in NE progression and are highly expressed in metastatic tumors [169]. Hypoxia was also implicated in development of NE by downregulation of Notch signaling [309].

Molecular changes strongly associated with NEPC were identified by NGS of RNA, and most frequent are overexpression of EZH2 and amplification of Aurora kinase A and NMYC [310]. Concurrent amplification of NMYC and AURKA is strongly associated with NEPC [311], and is also a frequent feature of the neuroendocrine childhood tumor neuroblastoma. AURKA is necessary for the growth of MYCN amplified neuroblastoma providing an essential function in stabilization of NMYC protein [312].

The neuroendocrine phenotype in PCa was shown to be mechanistically linked to the downregulation of transcriptional complex REST [313]. REST is expressed in neural stem cells and is known as a transcriptional repressive complex that recruits HDACs, and is essential for the maintenance of the stem cell phenotype [314, 315] and suppression of neuronal phenotype. In addition to downregulation of REST, a component of the REST, PHF21A was found to be differentially spliced in NEPC to produce a protein lacking DNA binding domain and therefore rendered inactive in respect to its function [313]. Finally, a recent report describes loss of RB1 as a common occurrence in CRPC with neuroendocrine features [316].

In addition to classic chemotherapy regimens, potential novel treatment options for this clinically aggressive type of PCa could involve Aurora kinase inhibitors. Clinical trials with inhibitor MLN8237 are ongoing (NCT01799278) or planned (NCT01848067), in combination with abiraterone or chemotherapy. A recent study performed *in vitro* and in a mouse neuroblastoma model demonstrated that inhibition of AURKA with MLN8237 or MLN8054 actually triggers degradation of NMYC mediated by the Fbxw7 ubiquitin ligase [317].

### Diagnostic and prognostic biomarkers

Search for diagnostic and prognostic tests in localized PCa. Testing for PSA prostate-specific antigen as a screening tool for PCa has been useful in diagnosis and follow-up to treatments in PCa, but it has shortcomings. These include false positives, unnecessary treatments for men with low grade PCa but elevated PSA, and occasional lack of PSA in high grade PCa (particularly with NE phenotype). Inter-individual variations in PSA levels have been reported to be associated with three particular polymorphisms in the individual genomes [318], and this information might be used for “correction” of PSA scores.

Detection of TMPRSS2-ERG (T/E) fusion in urine in combination with serum PSA was reported to be successful in risk stratification for PCa [319]. A combination urine test for ERG and PCA3 (a noncoding RNA associated with PCa) by PCR, and PSA serum levels, was reported to have a superior diagnostic value compared to either marker alone [320], in particular in the active surveillance group of patients [321]. Obviously, considering that T/E fusion is associated with 40 to 60% of PCa, these tests will not be useful for T/E negative patients. These tests, however, have not entered routine clinical use.

The predictive value of testing is particularly high in active surveillance, a treatment (or lack of treatment) approach that has been supported by several recent clinical trials. They demonstrated better quality of life for low-risk prostate cancer patients who were actively monitored rather than treated for their disease. One such study, the Prostate Cancer Intervention Versus Observation Trial (PIVOT), found that men who have low-risk cancer may not need early treatment for prostate cancer [322]. However, the prognostic markers are badly needed to identify patients who will benefit from aggressive treatments versus the truly low-risk group. PCA3, a noncoding RNA overexpressed in PCa [323], is one of the biomarkers explored, but it could be useful only in combination with testing for ERG [324, 325]. Both could be detected in urine, which is an important consideration. A much more extensive test, Prolaris from Myriad, analyzes an expression signature of 31 cell-cycle related genes to predict biochemical recurrence, but tumor biopsies are needed for this analysis. Genomic Health will be providing the OncotypeDX test soon to help identified patients in danger of being “overtreated”. Both of these tests hold promise but their clinical validation is not yet complete.

A recent study has reported identification of a 19 gene expression signature enriched in genes associated with aging and senescence, which allows to distinguish indolent low Gleason tumors. Moreover, expression of just three genes: FGFR1, PMP22, and CDKN1A accurately predicted outcome of low Gleason score tumors. Protein expression of this three-gene panel in biopsy samples distinguished Gleason 6 patients who failed surveillance over a 10-year period [326], but these tests need a full validation.

ConfrimMDx test from MDxHealth examines methylation status of a three genes, GSTP1, APC and/or RASSF1 in PCa biopsies. The company claims that their test is more accurate in detection of PCa in biopsies, because it could detect the effects of cancerous growth in cells adjacent to it, and does not have to rely on the identification of PCa foci in the needle core biopsies.

Prognostic tests for CRPC. Significant work has been conducted in order to develop a minimally invasive diagnostic procedure, considering the risks associated with needle biopsies and the fact that biopsies of metastases present a number of risks and limitations. “Liquid” biopsies or predictive gene signatures based on DNA and mRNA analyses of whole blood are being developed. In one study, an expression signature of six genes was highly effective in predicting survival [327], and, similarly, a nine gene signature was highly predictive in another study [328], but these are relatively far away from clinical implementation.

Isolation and analysis of circulating tumor cells (CTC) is a developing technology that is promising in metastatic cancers. According to several studies, the mere enumeration of CTCs in blood samples is prognostic and could be predictive of response to therapies in CRPC [329-332]. Capture of CTC presents a technological challenge, such as the frequent EMT observed in CRPC, which eliminates the expression of epithelial markers (antibodies to E-cadherin are frequently used to selectively isolate circulating metastatic cells from whole blood.) Identification of cell surface markers selectively expressed on metastatic cells with stem cell/EMT signature is needed. The challenges and significance of CTC analyses in PCa were reviewed recently [333, 334].

Reactivation of AR signaling despite continuous treatment with new drugs such as abiraterone (CYP17A1 inhibitor) is a common phenomenon. Prediction of response in patients is highly desirable, and a non-invasive test is much preferred to repeated biopsies. Isolation and analysis of CTC was explored as a diagnostic or prognostic factor for ADT. Earlier attempts to analysis of CTC were limited to quantification only. The feasibility of measuring the AR pathway activity in CTC was demonstrated [335]. This approach became feasible due to technological advances in microfluidic capture of CTC and imaging, enabling single cells immunofluorescence analysis of AR activity. The “AR-ON” signature was observed in untreated patients whereas patients with CRPC had mixed levels of AR activation on, off and mixed). First line ADT induced a switch from AR-ON to AR-OFF, but secondary hormonal therapy evoked mixed responses. Responses to second line ADT (abiraterone) showed presence of “AR-mixed” CTCs and increasing “AR-on” cells, which were associated with an adverse treatment outcome. This test could be used a predictive of responses to ADT.

Other possibilities for novel non-invasive tests include mRNA seq in captured CTC [336] and detection of telomerase hTERT mRNA in plasma [337]. The latter was reported to be a useful predictor of biochemical recurrence, and could be considered in combination with other known markers.

### Immunotherapy for PCA

There has always been an interest in development of immunotherapeutics for PCa, and the only approved cell-based immunotherapy, Sipuleucel-T, was developed for PCa. Numerous other approaches are in clinical development (Table 4), some of which are mentioned below.

**Table 4:** Targeting immune system in prostate cancer

Approach	Agent description	Drug	Stage of development
			
Blockade of the inhibitory T cell receptor CTLA4	Antibody to CTLA4 expressed on immune cells	Ipilimumab/Yervoy	In numerous clinical trials; part of combination therapies.
Blockade of the inhibitory T cell receptor PD-1	Antibody to PD-1	CT-011/Pidilizumab	One trial with CT-011 in combination with Sipuleucel-T, phase 2
Vaccination	Fowlpox virus based vaccine; expression of immunostimulants B7.1, ICAM-1, and LFA-3 and PSA	PROSTVAC®-VF	In clinical trials; phase II; in combination therapies with other agents
Cell based immune therapy	Enriched for dendritic cells (exposed to GM-CSF) fused to prostatic-acid phosphatase (PAP) DC expressing PSMA Autologous T cells expressing CAR to PSMA	Sipuleucel-T BPX-201 T cells with CAR	Approved for minimally symptomatic metastatic CRPC, 2010 Phase 1 Phase I
Whole cell vaccination	Irradiated PCa cells expressing GM-CSF	GVAX	Phase 1,2; combination
Activation of co-stimulatory receptor	Antibody to OX40, stimulatory	Anti-OX40	Phase 1,2; combination treatment

#### 

##### Immunomodulatory antibodies

A growing number of trials are ongoing with the immune checkpoint antibodies in prostate cancer. Ipilimumab, FDA approved anti-CTLA4 antibody, is in several trials in PCa, including randomized phase III NCT01057810 for patients with asymptomatic mCRPC and randomized phase III trial NCT00861614 with ipilimumab or placebo administered after radiotherapy. The results of latter trial failed to reach the primary endpoint of increasing OS, but showed some signs of activity of ipilimumab that warrant further investigation [338]. The second study of ipilimumab which examines the drug in chemotherapy-naïve patients, is still under way. Ipilimumab is also combined with Abiraterone and prednisone in a phase II study NCT01688492 for patients with progressive mCRPC. Phase II trial NCT01498978 is exploring addition of ipilimumab to patients with mCRPC under treatment with ADT agents such as LHRH agonists or antiandrogens such as bicalutamide. Ipilimumab is also being evaluated in a neoadjuvant setting (phase II NCT01194271), and in combination with Sipuleucel-T in phase II NCT01832870, as well as several other trials.

In spite of successes achieved with anti-PD-1 and PD-L1 antibodies in other malignancies, there are only two clinical trials with these agents ongoing for prostate cancer. NCT01420965 combines sipuleucel and anti-PD-1 antibody CT-011, and anti-PD-L1 antibody MSB0010718C is tested in a several cancer types including prostate. The relative dearth of trials with checkpoint antibodies is most likely due to the fact that in the trial of BMS-936558, anti PD-1 antibody, remarkable responses were observed in patients with melanoma, NSCLC and RCC, but not PCa [339]. Immunostimulatory antibody to OX-40 is in early clinical testing (NCT01303705), but will be administered only on short-term basis after cyclophosphamide. The antibody is of mouse origin and cannot be used for longer treatments.

##### Adoptive cell transfer

Phase I trial NCT01140373 for mCRPC patients is testing harvested autologous T cells transduced *in vitro* with CAR (chimeric antigen receptor) recognizing PSMA. Treatment will involve myeloablation with cyclophosphamide in patients with CRPC.

An early phase clinical study is exploring potential of natural killer (NK) cells in various malignancies including PCa. In particular, this phase I trial (NCT00720785) will examine if the limited anti-tumor activity of NK cells could be significantly increased by pretreating patients with proteasome inhibitor bortezomib, which has been reported to enhance the sensitivity of tumor cells to NK killing in numerous studies [340, 341]. In the future, the complex regulation of NK cells activity by tumors themselves will have to be considered. A very recent study has demonstrated a striking role of the well known NK ligand NKG2D in regulating the cytotoxic activity of NK cells in prostate tumors in a mouse model. Apparently, membrane-restricted and soluble NKG2D ligands pose opposite impacts on tumor progression and metastasis. The membrane-restricted NKG2D ligand MICB.A2 could sustain NKG2D protective immunity and prevent spontaneous tumorigenesis, whereas the native NKG2D ligand MICB facilitates tumor progression through soluble ligand-mediated impairment of NK cell peripheral maintenance [342].

##### Vaccination

PROSTVAC-V and PROSTVAC-F are vaccinia and fowlpox based virus vaccines expressing PSA and TRICOM (three immunostimulatory proteins B7.1, ICAM-1, and LFA-3), and with GM-CSF. PROSTVAC-V is given for priming, and PROSTVAC-F for boosting the response. A phase II trial was completed without clear clinical benefits, such as increase in PFS, observed. However, the clinical evaluation of PROSTVAC-V/F should be re-considered considering different endpoints for immune therapies [343]. Indeed, evaluation of patients at three year post-study showed an increase in OS [344]. Phase III trial NCT01322490 for PROSTVAC-V/F with GM-CSF is in progress. Phase I trial NCT00450463 examines PROSTVAC-V/F-TRICOM versus placebo in patients treated with flutamide, and phase II NCT01875250 with enzalutamide. Development of a humoral response to an viral antigen in PROSTVAC as reported to be a potential predictive marker for favorable response to PROST-VAC in patients [345].

Phase II trial NCT01341652 examines PAP vaccine plus GM-CSF versus GM-CSF alone in non-metastatic PCa. Adenovirus/PSA vaccine is tested in Phase II NCT00583024 in hormone-refractory PCa, while NCT00583752 will test the same vaccine in men with locally treated PCa.

##### Dendritic cells

Sipuleucel-T/Provenge/ was the first cellular immunotherapeutic to be approved by the FDA to treat cancer. This treatment consists of autologous peripheral blood mononuclear cells (PBMCs) enriched for a CD54+ DCs (dendritic cells). These are primed *in vitro* with the recombinant fusion protein consisting of prostatic acid phosphatase (PAP) and GM-CSF. This causes the activation and expansion of the autologous antigen-presenting cells (APCs) and lymphocytes, even though the precise mechanism is still unknown. Treatment with Sipuleucel-T does not have an effect on levels of PSA or radiological parameters of disease, but has a modest effect on OS.

Sipuleucel-T is also examined as a neoadjuvant in patients with localized PCa: phase II, NCT00715104; in combination with external beam radiation therapy in CRPC patients (NCT01807065, Phase II), and with abiraterone in phase II NCT01487863. Combination of Sipuleucel-T with Ipilimumab, an immune checkpoint antibody targeting inhibitory CTLA-4, is in a phase II trial NCT01804465 which examines immediate versus delayed CTLA-4 blockade, and in phase I NCT01832870 for advanced PCa. A trial of Sipuleucel-T with another checkpoint antibody, CT-011 targeting PD-1 is also ongoing (NCT01420965, phase II).

BPX-201 DCs vaccine with activating agent AP1903 is undergoing testing in phase I trial NCT01823978 for mCRPC. DCs in this trial are transduced with adenovirus-based vector expressing PSMA and a fusion protein composed of synthetic inducible adjuvant iMC, drug-inducible costimulatory CD40 receptor (iCD40) and the adaptor protein MyD88, with potential immunomodulating and antineoplastic activities. The iCD40 contains a membrane-localized cytoplasmic CD40 domain fused to the FK506 modified drug-binding protein 12 (FKBP12). Upon intradermal administration of BPX-201, these DCs accumulate in local draining lymph nodes. Twenty-four hours after vaccination, the dimerizing agent AP1903 is administered. AP1903 binds to the drug binding domain, leading to iMC oligomerization and activation of iCD40 and MyD88-mediated signaling in iMC-expressing DCs.

### New directions

#### 

##### Neoneurogenesis in PCa development

An exciting new target in treatment of PCA is neoneurogenesis, or the ingrowth of new nerve endings into a tumor. It was discovered recently that the autonomous nervous system plays a direct role in PCa growth and metastasis. Sympathetic and parasympathetic nerves in the normal prostate control the physiological function of both muscle fibers and epithelial compartment, but their involvement in PCa was unsuspected. As was demonstrated in [346], adrenergic fibers from the sympathetic nervous system contribute to the development of PCa by release of noradrenaline which stimulates β2- and β3-adrenergic receptors expressed on smooth muscle cells in the stroma. Deletion of β2- and β3-adrenergic receptors in stroma prevented development of PCa in different mouse models of PCa. Cholinergic fibers of the parasympathetic nervous system (PNS) stimulate dissemination of prostate cancer cells by releasing acetylcholine that stimulates muscarinic receptors on stromal cells. Deletion of type 1 muscarinic receptors in stroma inhibited tumor invasion and metastasis. Importantly, higher overall densities of nerve fibers were detected in PCa patients with poor prognosis compared to a group with better prognosis. Targeting the autonomous nervous system could therefore prevent tumor progression in PCa.

##### Metabolic regulation of PCa development

A recent population study performed in Toronto showed that increasing duration of metformin use among diabetic men after a diagnosis of prostate cancer was associated with decreased prostate cancer–speciﬁc and all-cause mortality [347]. The findings were significant irrespective of what treatments the subjects were receiving for their PCa. Metformin is a widely used drug to treat type II diabetes, and is currently explored in numerous types of cancer, including PCa, in about 10 trials. In particular, addition of metformin to various forms of ADT is explored based on the rationale that ADT is associated with the metabolic syndrome, hyperinsulinemia and insulin resistance. Hyperinsulinemia was reported to stimulate tumor growth and development of CRPC via activation of IGFR. Metformin through its activation of the AMPK-LKBI pathway reduces liver gluconeogenesis secondarily decreasing insulin levels, which might explain the effects on tumor growth. Other effects of metformin on cellular metabolic processes could contribute to its anti-cancer properties, such as indirect effects on mTOR and SIRT1. Metformin acts directly on mitochondrial complex I reducing respiration rates, and this activity is probably relevant to the recently demonstrated improvement of prostate tumors oxygenation and radiotherapy response *in vivo* [348]. Metformin use was associated with significant decrease in biochemical relapse in patients [348].

L-type amino acid transporters (LATs) uptake neutral amino acids including L-leucine into cells, stimulating mTORC1 signaling. LAT1 and LAT3 are overexpressed in PCa, and they are responsible for increasing nutrients and stimulating cell growth. LAT3, in particular, is expressed at high levels in all stages of PCA, and its expression is suppressed after ADT [349]. Pharmacological inhibition of LATs lead to downregulation of the E2F regulated M phase genes, and silencing of LAT1 or LAT3 in the xenograft model inhibited tumor growth and metastases. [349]

##### Regulation of AR activity by long noncoding RNA (lncRNA)

A recent study uncovered how two lncRNAs, PRNCR1 and PCGEM1, overexpressed in many CRPCs, concordantly enhance AR transcriptional activity in a complicated series of events. These lncRNAs localize to chromatin in androgen response area, whereby PRNCR1 binds the acetylated carboxyl end of AR, recruits methylase DOTL1, which methylates the amino terminus of AR, a pre-requisite to binding of PCGEM1. AR-bound PCGEM recruits protein Pygo2 bound to H3K4me3 on chromatin in the promoter area, thus inducing chromatin looping that brings enhancer and promoter area into close proximity. The proximity of AR bound enhancer and promoter sequences of target genes results in enhanced transcription of AR target, many of which contribute to oncogenesis [350]. These lncRNAs could serve as therapeutic targets because their silencing inhibited growth of xenograft tumors.

##### Potential role of HHV-8 in prostate carcinogenesis

Presence of HHV-8 (also known as Kaposi sarcoma herpes virus) in normal prostate, prostate cancers and biological fluids was reported in a number of publications, some of which documented higher prevalence of HHV-8 in prostate cancer or seropositivity for HHV-8 in PCa patients, while others reported no such association. In general, HHV-8 infection was not linked etiologically to PCa. However, a recent publication reported that HHV-8 infection of androgen-responsive PCa cells confers androgen-independent growth via activation of EZH2 controlled gene silencing [351]. These findings warrant further investigation of the role that HHV-8 might play in development of CRPC.

## CONCLUDING REMARKS

Even though NGS studies were conducted for prostate cancer, the identification of well defined and clinically meaningful subtypes based on genomic profiling has been difficult. It is likely due to the high number of molecular alterations that contribute to the development of localized PCa. For example, about half of prostate cancers have translocations involving the ETS family members, but these are not sufficient to cause frank PCa. The additional alterations that cooperate with deregulated ETS are many, and presumably each of them (for example, loss of PTEN versus loss of TP53 function) could determine the precise clinical subtype of the emerging tumor. The other known drivers of the PCa early development (in fusion-negative cancers) are also several: SPOP mutation, SPINK overexpression, CHD1 deletion and TAK1 loss, as well as chromosomal losses not characterized in terms of genes involved. These molecular subtypes of PCa await analysis of the clinical significance of the underlying somatic changes.

The pervasive involvement of AR signaling in the later stages of PCa presents a conundrum as well, because it affects an entire massive transcriptional program that is further affected by additional alterations in tumor suppressors, transcription factors, chromatin remodeling enzymes and, almost universally, developmental factors. These multiple perturbations in advanced PCa present great difficulties in terms of not only treatments, but also identification of biomarkers of risk or prognostic/theranostic value.

Since androgen regulated pathways are affected in the vast majority of advanced PCa, the ADT is a logical therapeutic intervention. The persistent significance of the AR signaling in CRPC was recently validated by the evidence of the clinical efficacy of androgen synthesis inhibitors (abiraterone) and the novel, second-generation AR antagonists (enzalutamide). However, ADT is often used under circumstances where the mechanisms of resistance to it are most likely already present in a subpopulation of PCa cells. One possibility is the existence of a compartment of the androgen-independent cells with characteristics of stemness that already have the driver mutations such as ETS translocation or PTEN loss, and that are propelled into reproduction and acquisition of further genetic changes by the drop in androgen levels. Alternatively, a population of androgen independent or almost independent cells might emerge as a consequence of the ADT by selecting rare cells with *de novo* alterations in other signaling, developmental or epigenetic pathways that bypass the need for androgen signaling.

The universal development of resistance to ADT, including the newest agents, is a testimony not only to the adaptability of the activity of AR pathway to the low or even absent androgens, but also an evidence of enormous adaptability of PCa cellular oncogenic pathways. Resistance to ADT is enormously important clinically, and is the subject of intense research (reviewed in [135, 352, 353]. Emergence of CRPC subtypes (like NEPC) that do not express AR and therefore are truly independent of androgen signaling presents a clinical conundrum that warrants more research in treatment options.

It is becoming a shared understanding that in the future, ADT will be administered as one component in a combination of therapies, to try and forestall the development of resistance. However, the real conundrum will be to find a right “partner” to the ADT. The problem lies in the identification of the particular pre-existing or emerging alterations that ultimately contribute to the resistance to ADT. The acknowledged role of the tumor heterogeneity in this process is hard to dispute. However, it is difficult to address it in the context of limited biopsies, even if several are taken from the tumor. Comprehensive genomic analysis of the CTCs or free circulating tumor DNA might be a step toward identification of mutations present in heterogeneous subpopulations of tumor or its metastases.
